# Myeloid cell MHC I expression drives CD8^+^ T cell activation in nonalcoholic steatohepatitis

**DOI:** 10.3389/fimmu.2023.1302006

**Published:** 2024-01-11

**Authors:** Victoria R. Adams, Leonard B. Collins, Taufika Islam Williams, Jennifer Holmes, Paul Hess, Hannah M. Atkins, Grace Scheidemantle, Xiaojing Liu, Mareca Lodge, Aaron J. Johnson, Arion Kennedy

**Affiliations:** ^1^ Department of Molecular and Structural Biochemistry, NC State University, Raleigh, NC, United States; ^2^ Molecular Education, Technology and Research Innovation Center (METRIC), NC State University, Raleigh, NC, United States; ^3^ Department of Chemistry, NC State University, Raleigh, NC, United States; ^4^ College of Veterinary Medicine, NC State University, Raleigh, NC, United States; ^5^ Center for Human Health and Environment, NC State University, Raleigh, NC, United States; ^6^ Division of Comparative Medicine, UNC Chapel Hill, Chapel Hill, NC, United States; ^7^ Department of Immunology, Mayo Clinic, Rochester, MN, United States

**Keywords:** liver, NASH, fibrosis, H2Kb, CD8^+^ T cells, immunopeptidome

## Abstract

**Background & aims:**

Activated CD8^+^ T cells are elevated in Nonalcoholic steatohepatitis (NASH) and are important for driving fibrosis and inflammation. Despite this, mechanisms of CD8^+^ T cell activation in NASH are largely limited. Specific CD8^+^ T cell subsets may become activated through metabolic signals or cytokines. However, studies in NASH have not evaluated the impact of antigen presentation or the involvement of specific antigens. Therefore, we determined if activated CD8^+^ T cells are dependent on MHC class I expression in NASH to regulate fibrosis and inflammation.

**Methods:**

We used H2Kb and H2Db deficient (MHC I KO*)*, Kb transgenic mice, and myeloid cell Kb deficient mice (LysM Kb KO) to investigate how MHC class I impacts CD8^+^ T cell function and NASH. Flow cytometry, gene expression, and histology were used to examine hepatic inflammation and fibrosis. The hepatic class I immunopeptidome was evaluated by mass spectrometry.

**Results:**

In NASH, MHC class I isoform H2Kb was upregulated in myeloid cells. MHC I KO demonstrated protective effects against NASH-induced inflammation and fibrosis. Kb mice exhibited increased fibrosis in the absence of H2Db while LysM Kb KO mice showed protection against fibrosis but not inflammation. H2Kb restricted peptides identified a unique NASH peptide Ncf2 capable of CD8^+^ T cell activation *in vitro*. The Ncf2 peptide was not detected during fibrosis resolution.

**Conclusion:**

These results suggest that activated hepatic CD8^+^ T cells are dependent on myeloid cell MHC class I expression in diet induced NASH to promote inflammation and fibrosis. Additionally, our studies suggest a role of NADPH oxidase in the production of Ncf2 peptide generation.

## 1 Introduction

Nonalcoholic fatty liver disease (NAFLD) represents a spectrum of liver pathologies, beginning with hepatic steatosis progressing to nonalcoholic steatohepatitis (NASH) and even hepatocellular carcinoma (HCC) (1). NASH is characterized by immune cell infiltration, inflammation, oxidative stress, and fibrosis in the liver (2). Recent studies have demonstrated in obese models of NASH, a pathogenic subset of hepatic CD8^+^ T cells that are elevated and regulate hepatic inflammation and fibrosis (3–8). Although liver fibrosis was thought to be irreversible, recent studies have shown NASH associated fibrosis can be resolved and is dependent on a protective subset of CD8^+^ T cells during resolution in mice (9). Although studies have highlighted various subsets of CD8^+^ T cells involved in both NASH development and resolution, further investigation is required to better understand mechanisms that regulate immune cell activation.

In both humans and mouse, NASH-induced B and T cell infiltration positively correlate with the presence of antibodies targeting antigens derived from oxidative stress (10). Oxidative stress in the liver is especially detrimental as it can play a role in cellular dysfunction, injury, and even cell death (11–13). With the advancement of single cell RNA sequence, current studies have identified multiple subsets of CD8^+^ T cells in human and mouse models of NAFLD (9, 14). Resident pathogenic CD8^+^ T cells in NASH are classified as auto-aggressive toward hepatocytes. This subset is characterized by expressing high levels of CXCR6, cytotoxicity (granzyme), and exhaustion marker PD-1. However, the hepatic CD8^+^ T cells were discovered to act in an antigen-independent manner relying on IL-15 driven transcriptional reprogramming and metabolic signals acetate and ATP for activation (14). Additionally, a subset of CD8^+^ T cells during NASH resolution operate in a CCR5-dependent chemoattractant manner also relying on IL-15 (9).

Major histocompatibility complex (MHC) class I molecules are expressed on all nucleated cells and are responsible for presenting short antigen peptides (8-11 amino acids in length) to CD8^+^ T cells. In mice, this region is referred to as the histocompatibility 2 (H2) complex and expresses three class I loci (K, D, and L) equivalent to human class I loci HLA-A, HLA-B, and HLA-C. In humans, limited studies have addressed the impact of MHC Class I in regulating NAFLD associated pathologies. Human HLA class I alleles such as HLA*B27 are associated with advanced steatosis, while alleles HLA*C4, HLA*A31 and HLA*C6 correlate with NASH and advanced fibrosis (15, 16). These genes are highly polymorphic, having numerous alleles for each locus (k, d, b, k, q), and are involved in regulating the peptide binding activities of MHC I (17). Peptides are obtained through proteasomal degradation of ubiquitinated proteins where they are loaded onto MHC I molecules in the ER and brought to the cell surface. They are typically presented on classical antigen presenting cells (APCs) such as macrophages, monocytes, or dendritic cells. At the cell surface CD8^+^ T cells are primarily activated by MHC class I bound peptides. Each T cell receptor has the capability for rearrangement which allows these cells to recognize many different peptides in order to become activated (8). However, it remains to determined how the MHC I immunopeptidome is altered during NASH and if this plays a role in CD8^+^ T cell activation. Thus, understanding the role of peptide presentation by MHC class I is important for elucidating mechanisms of CD8^+^ T cell activation in NASH.

Our findings indicate that CD8^+^ T cell activation in NASH is dependent on H2Kb expression. Knockout of H2Kb and H2Db leads to significant reductions in CD8^+^ T cell activation and hepatic fibrosis during NASH development. Lack of H2Kb in myeloid cells protected against liver fibrosis and CD8^+^ T cell activation but not inflammation. Mice with NASH expressed a unique hepatic H2Kb immunopeptidome compared to steatosis and normal mouse. We identified the NASH peptide Ncf2 and demonstrated this peptide activates NASH CD8^+^ T cells *in vitro*. Activated Ncf2 specific CD8^+^ T cells were also detected in NASH mice *in vivo* This is further supported by the absence of the Ncf2 peptide during fibrosis resolution. Thus, the Ncf2 peptide may be a driving factor in antigen dependent CD8^+^ T cell activation in NASH.

## 2 Materials and methods

### 2.1 Animal models

Male 5-wk-old C57BL/6J and low-density lipoprotein receptor knockout (LDLRKO) mice were originally purchased from Jackson Laboratories (Bar Harbor, ME) and further propagated in our colony. C57BL/6J mice were used to generate MHC I-deficient mice that lack endogenous H2Db and H2Kb (MHC I KO). Transgenic Kb mice were generated by introducing the H2Kb transgene (Kb LoxP) into MHC I KO mice developed by the Mayo Clinic Transgenic Mouse Core (Rochester, MN). Kb mice were then crossed with MHC I deficient mice expressing Cre recombinase under the LysM promoter (LysM Kb KO, myeloid cell specific). This cross generated a conditional knockout of Kb in myeloid cells. All Mouse genotypes were confirmed through flow cytometry. MHC I KO, Kb, and LysM Kb KO mice were donated from Dr. Aaron Johnson and further propagated in our colony (18).

*WT Amylin NASH Model*. Male 6-wk-old C57BL/6J (WT) mice were fed chow or amylin diet (AMLN, 40 kcal% fat, 20 kcal% fructose and 2% cholesterol, Diet # D09100310i, Research Diets) for 28 weeks. *LDLRKO NASH model*. Male 6-wk-old *LDLRKO* mice were fed chow or western diet (WD, 42 Kcal% fat with 0.2% added cholesterol, TD.22137; Harlan Laboratories) for 12 weeks. *WT Sucrose WD NASH model*. Male 6-wk-old C57BL/6J wild type (WT) mice were fed chow or western diet (WD, 42 Kcal% fat with 0.2% added cholesterol, TD.22137; Harlan Laboratories) with 30% glucose/fructose water for 25 weeks. *Taconic Amylin NASH Model*. C57BL/6NTac (Tac) male mice were purchased from Taconic Biosciences on chow or amylin diet (AMLN, 40 kcal% fat, 20 kcal% fructose and 2% cholesterol, Diet # D09100310i, Research Diets) remaining on diet for a total of 28 weeks. *Taconic Amylin Resolution Model*. Taconic mice were purchased from Taconic Biosciences on chow or amylin diet as described previously. For the resolution model (RES), NASH mice after 28 weeks on amylin diet were switched to a chow diet for 5 weeks. *MHC I KO, Kb, and LysM Kb KO Studies.* 6-wk-old male mice from WT, MHC I KO, Kb, LysM Kb KO were fed chow or amylin diet (AMLN, 40 kcal% fat, 20 kcal% fructose and 2% cholesterol, Diet # D09100310i, Research Diets) for 28 weeks.

All Mice were housed with ad libitum access to food and water on a 12-hour light/dark cycle. Mice were sacrificed between the ages of 15-34 weeks. Tissues were snap-frozen and stored in -80°C freezer. All animal procedures were approved by the Institutional Animal Care and Use Committee (IACUC) at North Carolina State University under protocol 21-502-B.

### 2.2 Immune cell isolation from liver

Mice were anesthetized and perfused through the heart with 1X PBS. Mouse livers were collected and minced in RPMI, 1 mg/ml Collagenase IV, 2 mg/ml Collagenase II, 1 mg/ml Protease, and 0.01 mg/ml DNase and incubated at 37°C for 25 min shaking. The cell suspension was filtered through a 100-µm filter with wash buffer and centrifuged at 443 xg for 6 min at 4°C. Supernatant was discarded and the pellet washed and centrifuged at 443 xg for 6 min at 4°C. The pellet was resuspended in 33% Percoll and centrifuged at 850 xg for 15 min at 4°C with minimum break and accelerator. The pellet was resuspended in wash buffer and centrifuged at 300 xg for 5 min at 4°C and the supernatant discarded. The pellet was resuspended in ACK lysing buffer and incubated at room temperature for 5 min. After incubating, wash buffer was added and centrifuged at 300 xg for 5 min at 4°C. The supernatant was discarded and the pellet resuspended in FACS buffer filtered through a cell strainer cap tube and prepared for cell culture or flow cytometry.

### 2.3 CD8^+^ T cell isolation from spleen

Mouse spleens were isolated and strained through a 100-µm filter with FACS buffer and centrifuged at 500 xg for 5 min at 4°C. Pellets were washed with FACS and centrifuged at 500 xg for 5 min at 4°C. Pellets were resuspended in ACK Lysing Buffer and incubated on ice for 5 mins. After incubation FACS buffer was added and centrifuged at 500 xg for 5 min at 4°C. Supernatants were discarded and pellets resuspended in 1ml FACS buffer and filtered through a cell strainer cap tube. Cells were centrifuged at 500 xg for 5 min at 4°C and resuspended in FACS buffer and prepared for T cell assays.

### 2.4 Flow cytometry

Isolated cells from the liver were incubated with Fc block followed by incubation with fluorophore conjugated antibodies on ice in FACS buffer for the following panels: T cell panel: CD8a (PE-Cy7, 1:200, BD Biosciences), TCRβ (APC-Cy7, 1:200, BD Biosciences), CD44 (A700, 1:200, BD Biosciences), and CD62L (APC, 1:200, BD Biosciences). T cell subsets panel: CD8a (PE-Cy7, 1:200, BD Biosciences), TCRβ (APC-Cy7, 1:200, BD Biosciences), CD44 (A700, 1:200, BD Biosciences), CD62L (APC, 1:200, BD Biosciences), CXCR6 (FITC, 1:100, Biolegend), CCR7 (PerCP-Cy5.5, 1:100, Biolegend), and Ncf2 Tetramer (PE, 1:100, NIH Tetramer Core Facility). NK T cell panel: NK1.1 (PE, 1:100, BD Biosciences), TCRβ (APC-Cy7, 1:200, BD Biosciences), CD8a (PE-Cy7, 1:200, BD Biosciences), and CD4 (AF488, 1:200, BD Biosciences). Macrophage Panel: F4/80 (A700, 1:200, Biolegend), CD64 (PE, 1:200, BD Biosciences), CD11b (FITC, 1:200, BD Biosciences), Ly6c (PerCP-Cy5.5, 1:200, BD Biosciences), H2Kb (APC-eFluor780, 1:200, ThermoFisher), and H2Db (PE-Cy7, 1:200, BD Biosciences). After incubation samples were washed twice with FACS buffer. Flow data was acquired on a Becton Dickinson LSRII machine in the NCSU flow Cytometry Core. All data was analyzed using FlowJo software v10.8. Flow gating strategies are provided (**Supplementary Figures 1A, B**).

### 2.5 Liver tissue histology staining

Paraffin-embedded sections from mouse livers were used for Hematoxylin and eosin (H&E) and Sirius red staining (7). Whole slide images were captured on BioTek Cytation 5 at 10X magnification.

### 2.6 RNA isolation and real time RT-PCR

For RNA isolation, 25 mg of frozen liver tissue was homogenized in Tri-Reagent (Fisher) and extracted using Direct-zol RNA MiniPrep kit (Genesee) according to manufacturer’s instructions. cDNA was synthesized on a BioRad iQ5 thermocycler using qScript cDNA supermix (QuantaBio) according to manufacturer’s instructions. Real-time RT-PCR analysis was performed using PerfeCTa qPCR FastMix II (QuantaBio) and TaqMan assay (ThermoFisher) on a 7500 fast Dx thermocycler. Relative gene expression was normalized to 18s expression and determined using the delta-delta CT method. All sample reactions performed in duplicate. All TaqMan assays used can be found in STAR methods.

### 2.7 Western analysis

A 50 mg piece of frozen liver was homogenized in 0.5 ml of RIPA buffer (150 mM Sodium Chloride, 1% Triton X-100, 0.5% Sodium Deoxycholate, 0.1% SDS, 50 mM Tris, and 1 mM EDTA) containing 1X protease and phosphatase inhibitor cocktail (Halt). Samples were centrifuged for 15 min at 12,000 rpm at 4°C. Supernatants were aspirated and protein quantified using Pierce BCA protein assay according to manufacturer’s instructions. Proteins were separated by SDS-PAGE and transferred to activated PVDF membranes. Membranes were blocked with OneBlock blocking buffer (Prometheus, Genessee Scientific) for 1 hr at room temperature shaking. Membranes were then probed with antibodies specific for Ncf2 (ThermoFisher), H2Kb (BioXCell), or H2Db (Santa Cruz) at 4°C shaking overnight. All blots were probed for β-actin (Cell Signaling Technology) 1 hr at room temperature. After blocking membranes were washed three times for 5 min in 1X PBS with 0.1% Tween-20 at room temperature. After washing membranes were incubated with Licor secondary antibodies goat anti-rabbit IRDye 800CW and goat anti-mouse IRDye 680RD at 1:10,000 dilutions with 0.1% Tween-20 for 1 hr at room temperature protected from light. Blots were washed again as described previously and imaged on Odyssey Infrared Imaging System. Band intensity quantification was obtained using Image Studio Lite version 5.2 software.

### 2.8 Isolation of H2Kb peptides from liver

Liver lysates were prepared following Kowalewski’s methods with some modifications (19). Livers were isolated and pooled together 5 Control or 5 NASH age matched mice or isolated individually and immediately minced in one volume of 2x solubilization buffer (1X PBS, 12% CHAPS, protease inhibitor mini tablet (Pierce), and PhosSTOP inhibitor tablet (Roche). Lysates were then manually homogenized and rinsed in 1X solubilization buffer and stirred for 1 hr at 4°C. Lysates were sonicated with 120w of ultrasonic power and 30% pulse length for 3 min on ice and then stirred for 1 hr at 4°C. Lysates were then centrifuged at 2,000 x g for 20 min. Supernatants were collected and subjected to ultracentrifugation at 150,000 x g for 70 min at 4°C. Supernatants were collected and filtered through a 0.22 µm pore syringe filter.

Column preparation was modified from methods by Chen and colleagues (20). 1 ml HiTrap NHS-activated HP immunoaffinity columns (Cytiva) were prepared based on the following steps. Briefly, a drop of ice-cold 1 mM HCl was added to the top of the column and 6 ml of ice-cold 1 mM HCl was added to the column at a flow rate not exceeding 1 ml/min. For antibody coupling 10 ml of 1 mg/ml anti-H2Kb mAb (Clone:Y3, BioXCell) antibody in antibody coupling buffer (0.2 M of NaHCO3, 0.25 M NaCl, pH 8.3) was circulated at a flow rate of 1 ml/min for 4 hr at 4°C with a peristalsis pump. To deactivate any excess active groups, the column was injected with 3 x 2 ml buffer A (0.5 M ethanolamine, 0.25 M NaCl, pH 8.3) and 3 x 2 ml buffer B (0.1 M sodium acetate, 0.25 M NaCl, pH 4) alternatively with a syringe not exceeding 1 ml/min. After the column was left to rest at room temperature for 30 min, alternate washes of 3 x 2 ml buffer B and 3 x 2 ml buffer A were injected into the column. The prepared liver lysate was then continually circulated over the column overnight at a flow rate of 1 ml/min at 4°C. Columns were then washed with the following buffers: 15 ml of wash buffer 1 (50 mM Tris-HCl, pH 8, 75 mM NaCl, and 1% CHAPS), 15 ml of wash buffer 2 (50 mM Tris-HCl, pH 8, 75 mM NaCl in deionized H2O), 25 ml of wash buffer 3 (50 mM Tris-HCl, pH 8, 225 mM NaCl in deionized H2O), and 35 ml of wash buffer 4 (50 mM Tris-HCl, pH 8 in deionized H2O). H2Kb peptides were eluted in 6 ml of 10% acetic acid and filtered using a 3kDa ultrafiltration filter (Millipore) and frozen in -80°C. Frozen peptide elutes were lyophilized overnight and resuspended in 300 µL of 0.1% TFA. Peptide solutions were desalted using peptide desalting columns (Pierce) according to manufacturer’s instructions.

### 2.9 Analysis of H2Kb peptides by LC-MS/MS

Immunoaffinity H2Kb captured peptide solutions were analyzed by a discovery proteomics workflow using a hybrid quadrupole-orbitrap mass spectrometer (Thermo Scientific™ Orbitrap Exploris™ 480, Bremen Germany) incorporating an Easy-Spray™ nanoelectrospray source (Thermo Scientific™, San Jose) coupled to an Easy-nLC™ 1200 nano-liquid-chromatography system (Thermo Scientific™, San Jose). Mass spectrometry data were acquired using non-targeted data-dependent acquisition (DDA) at top scan speed with a full experiment time of 3 seconds. Samples were injected in a random order. Commercially obtained, standardized bovine serum albumin (BSA) digest and HeLa digest were evaluated throughout the injection sequence to ensure proper nanoLC−MS/MS reproducibility. Proteins were identified by processing raw nanoLC/MS data with Proteome Discoverer 2.5 software (Thermo Scientific™, San Jose, CA) using a Mus musculus protein database obtained from Swiss-Prot (Taxon 10090 including sub-taxonomies; 17,090 sequences).

### 2.10 Analysis of lipid species by LC-MS/MS

Liver lipids were measured using methods previously described (21).

### 2.11 Peptide synthesis

Unique NASH H2Kb restricted peptides were synthesized at a crude purity from Peptide 2.0 Inc (Chantilly, VA). Synthetic peptides were used for functional assays and LC-MS/MS validation.

### 2.12 H2Kb peptide binding assay

RMA-S cells were seeded into a 96 well plate at 1x10^5^ per well in 100 µL of media (RPMI with 10% FBS) and incubated at 27°C for 18 hrs. Following incubation cells were treated with 100 µM of vehicle control, Ova, Ncf2 peptide, or Gpnmb peptide (FVYVFHTL) and incubated at 37°C for 5 hrs. Cells were harvested for flow cytometry and incubated with Fc block for 5 min followed by a 30 min incubation with fluorophore conjugated antibodies on ice in FACS buffer for H2Kb (APC-eFluor780, 1:200, ThermoFisher) and Propidium Iodide (PI, 1:10,000, ThermoFisher). Data for this assay was acquired on a BD Accuri C6 machine. All data was analyzed using FlowJo software v10.8.

### 2.13 CD8^+^ T cell activation assay

96 well plates were initially coated with IgG (clone RTK2758, BioLegend) and incubated at room temperature for 3 hr. Plates were washed twice with PBS and coated with 1 µg/ml anti-CD3 (clone 145-2C11, Bio-Rad) and 1 µg/ml anti-CD28 (clone E18, Bio-Rad) overnight at 4°C. Plates were washed twice with PBS prior to plating cells. Unstimulated samples were not cultured with CD3 or CD28 and supplemented only with IL-2. Immune cells were isolated from livers or spleens of NASH mice as described previously and CD8^+^ T cells were magnetically sorted using a CD8^+^ T cell isolation kit (Miltenyi Biotec). CD8^+^ T cells were stained with cell trace violet (Life Technologies) according to manufacturer’s instructions. RMA-S cells were treated with vehicle control, Ova, Ncf2 peptide, or Gpnmb peptide as described previously. CD8^+^ T cells were plated at 5x10^4^ per well and co-cultured with 1x10^4^ of treated RMA-S cells per well in a total of 200 µL of RPMI medium (Corning) supplemented with 10% FBS, L-glutamine (400 mM), penicillin (100 U/ml), streptomycin (100 µg/ml), 2-mercaptoethanol (50 µM), and IL-2 (100 ng). Cells were cultured at 37°C and harvested after 3 and 5 days and prepared for flow cytometry using the CD8^+^ T cell panel. T cell activation assay for genotype comparisons of WT, MHC I KO, and Kb CD8^+^ T cells were plated in 6 well plates with 1.5x10^5^ cells/well in 2 ml of media under stimulated or unstimulated conditions for 3 days and harvested for flow cytometry. Data for this assay was acquired on a Beckman Coulter CytoFLEX machine in the NCSU flow Cytometry Core. All data was analyzed using FlowJo software v10.8.

### 2.14 Cytokine analysis

Media from T cell activation assays were collected and analyzed using a 9-plex mouse luminex discovery assay targeting RANTES, Granzyme B, IFN-gamma, Il-2, IL-4, IL-6, IL-10, IL-13 and TNF alpha on the Bio-Rad Bio-Plex 200 multiplex suspension array system in the Advanced Analytical Core at UNC Chapel Hill. Media was diluted to 1:5 using assay buffer.

### 2.15 Statistical analysis

GraphPad Prism 10.0.2 software was used for all statistical analyses. Two-tailed unpaired Student’s t-tests were performed for two group comparisons. Two-way ANOVA was performed for genotype versus diet studies followed by multiple T test comparisons. All data is presented as the mean ± SEM. Data was considered statistically significant for P<0.05 (*), P<0.01 (**), P<0.001(***), and P<0.0001(****).

## 3 Results

### 3.1 H2Kb and H2Db is upregulated in myeloid cells NASH

Our previous work has shown increased activated CD8^+^ T cells in the LDLRKO obese/hyperlipidemia mouse model of NASH. We have shown that CD8^+^ T cells play a key role in regulating inflammation and fibrosis through CD8 antibody depletion and adoptive transfer studies (7). However, limited studies have focused on how these cells are activated and how antigen presentation plays a role in their activation (14).

**Figure 1:**
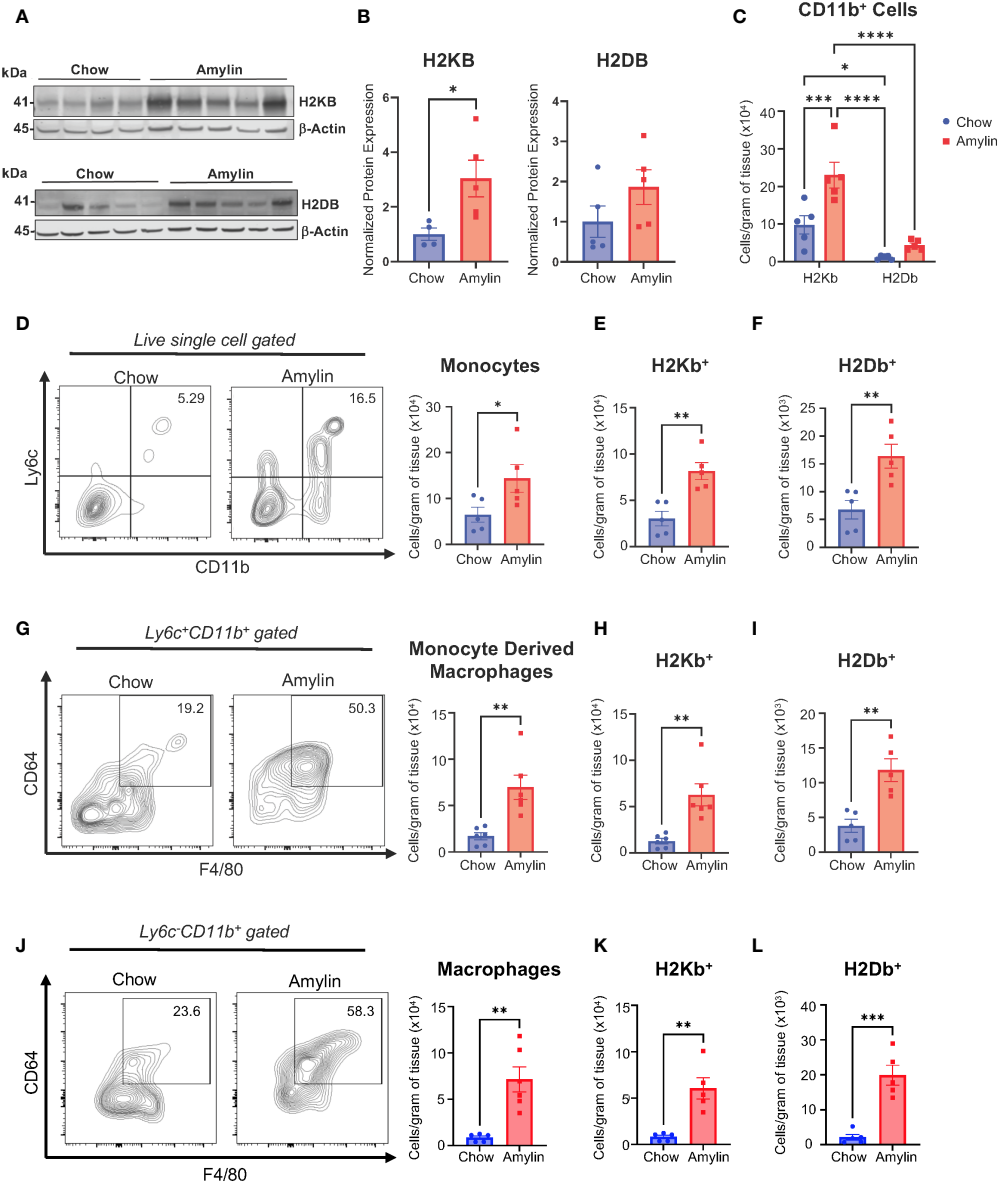
H2Kb is upregulated in myeloid cells in NASH. WT mice were fed chow or amylin diet for 28 wks (n=5 per group) in two replicate cohorts. **(A)** Total liver protein of H2KB and H2DB. **(B)** Quantification of protein expression normalized to β-actin. **(C)** Total CD11b^+^ cells gated for H2Kb and H2Db. **(D)** Flow analysis of monocytes (*Ly6c^+^CD11b^+^*) and monocyte H2Kb **(E)** and H2Db **(F)** expression. **(G)** Flow analysis of monocyte derived macrophages (*Ly6c^+^CD11b^+^CD64^+^F4/80^+^*) monocyte derived macrophage H2Kb **(H)** and H2Db **(I)** expression. **(J)** Flow analysis of macrophages (*Ly6^-^CD11b^+^CD64^+^F4/80^+^, CLEC4F^+^*) and macrophage H2Kb **(K)** and H2Db **(L)** expression. Flow plots show percentage of parent gate. Data shown as the mean ± SEM. Two-tailed unpaired Student’s t-tests and determined significant by P<0.05 (*), P<0.01 (**), P<0.001 (***), and P<0.0001(****).

We demonstrate that activated CD8^+^ T cells are significantly increased in obese mouse models of NASH using low density lipoprotein receptor knockout mice (LDLRKO) on western diet (WD) (**Supplementary Figures 2A, B**) and Taconic mice on amylin diet (Tac NASH) (**Supplementary Figures 2C, D**). Because CD8^+^ T cells are class I restricted, we evaluated the expression of MHC I isoforms, H2Kb and H2Db, under NASH conditions. Total liver protein analysis revealed H2KB protein expression was significantly upregulated during NASH with increasing trends for H2DB expression (**Figures 1A, B**, **Supplementary Figures 2E, F**). We used flow cytometry analysis to determine if NASH impacts myeloid specific expression of H2Kb and H2Db in WT mice fed amylin diet for 28 weeks. Macrophages and recruited monocytes are elevated in NASH and crucial to inflammation, fibrosis, and immune cell activation in chronic liver diseases (22). In CD11b^+^ myeloid cells, overall H2Kb expression was significantly increased compared to H2Db expression. Additionally, H2Kb expression was significantly increased in NASH compared to chow suggesting H2Kb may play a larger role in antigen presentation in NASH (**Figure 1C**). Our studies confirmed increased monocytes (*Ly6c^+^ CD11b^+^*), monocyte derived macrophages (*Ly6c^+^CD11b^+^F480^+^CD64^+^*), and macrophage populations (*Ly6c^-^CD11b^+^F480^+^CD64^+^*) in NASH (**Figures 1D, G, J**). Additionally, H2Kb and H2Db were significantly increased in the myeloid cell populations in NASH compared to chow controls (**Figures 1E, F, H, I, K, L**). These findings were consistent across Tac NASH and LDLRKO NASH models (**Supplementary Figures 3A–O**). In summary, H2Kb and H2Db are increased in myeloid cells in NASH livers. However, it remains unknown how changes in the expression of H2Kb or H2Db impact NASH progression.

### 3.2 Myeloid cell H2Kb controls NASH associated hepatic CD8^+^ T cell activation

Given that H2Kb had higher overall expression in myeloid cells compared to H2Db, we then investigated the impacts of H2Kb on the activation of CD8^+^ T cells in NASH. To probe the function of H2Kb in NASH, we used three genetically modified mouse models: mice lacking H2Kb and H2Db (MHC I KO), expressing only H2Kb and no H2Db (Kb), and conditional Kb knockout in myeloid cells (LysM Kb KO). By flow analysis, H2Kb and H2Db were not detected in the livers of MHC I KO mice. H2Kb was only detected with no H2Db expression in the Kb mouse model, and the LysM Kb KO mouse model was deficient in H2Kb on CD11b^+^ cells (**Supplementary Figures 4A–C**).

**Figure 2:**
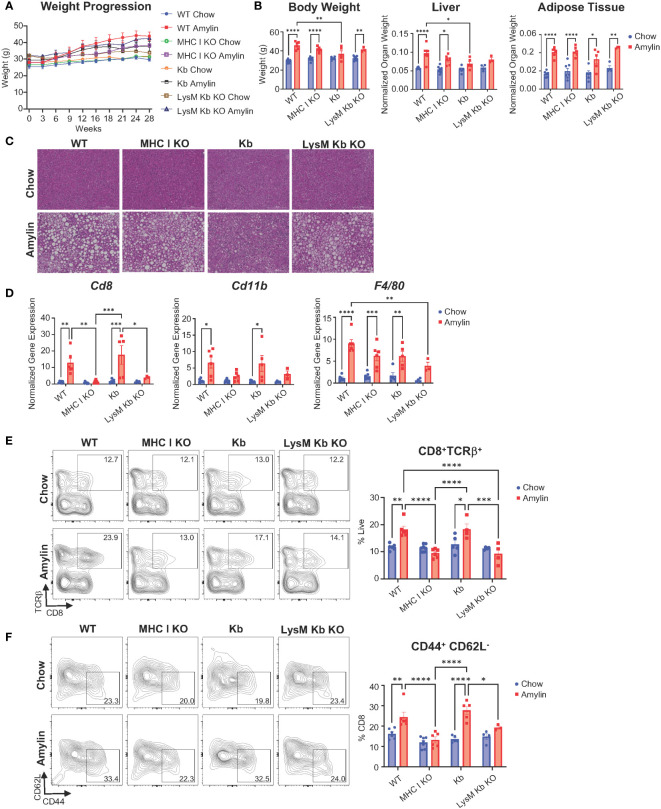
MHC I KO and LysM Kb KO prevent CD8^+^ T cell activation in NASH. WT, MHC I KO, Kb or LysM Kb KO mice were on diet for 28 wks (n=3-7 per group) in two replicate cohorts. **(A)** Weight progression over time. **(B)** Body weight, liver weight, and adipose tissue weight after 28 wks on diet. **(C)** Representative images of H&E staining of mouse liver sections. **(D)** Hepatic gene expression. Flow analysis of *Tcrβ^+^CD8^+^* T cells **(E)** and activation (*CD44^+^CD62L^-^*). **(F)** Flow plots show percentage of parent gate. Data shown as the mean ± SEM. Two-way ANOVA were performed and determined significant by P<0.05 (*), P<0.01 (**), P<0.001 (***), and P<0.0001 (****).

After 28 weeks, amylin diet significantly increased epididymal fat in WT, MHC I KO, Kb, and LysM Kb KO mice. MHC I KO mice on amylin diet behaved most similar to WT with increased body weight and liver weights. Interestingly, Kb and LysM amylin fed mice had reduced liver weights compared to WT amylin fed mice and no changes in body weight in the Kb amylin fed mice (**Figures 2A, B**). To further understand how MHC I impacts NASH pathology, liver tissue sections were stained with H&E. WT amylin mice demonstrated diffuse lipid accumulation and minimal lymphatic inflammation compared to chow controls. However, no significant changes were detected between WT, MHC I KO, and LysM Kb KO mice on amylin diet. In contrast, Kb amylin fed mice displayed hepatocyte necrosis and oval cell hyperplasia (**Figure 2C**, **Supplementary Table 1**). Notably, MHC I KO and LysM Kb KO mice were protected against NASH induced increases in hepatic gene expression of immune cell markers *Cd8* and *Cd11b.* Whereas Kb mice responded similar to WT mice on amylin diet with increased expression of both markers (**Figure 2D**). Next, we determined how the loss of MHC I, H2Kb expression only, and H2Kb expression in myeloid cells impacts hepatic CD8^+^ T cell activation during NASH development. Flow cytometry analysis demonstrated that MHC I KO and LysM Kb KO mice were protected from hepatic CD8^+^ T cell activation compared to WT and Kb amylin mice (**Figures 2E, F**).

Because MHC I disruption can reduce CD8^+^ T cell generation and impact natural killer (NK) T cell functions, we evaluated changes in CD8^+^ T cells and NK cells in WT, MHC I KO, Kb, and LysM Kb KO chow mice. Interestingly, no significant changes were identified in the number of liver CD8^+^ T cells between the WT and MHC I KO, Kb, or LysM Kb KO mice. However, MHC I KO mice demonstrated significant reductions in both spleen and blood CD8^+^ T cell numbers (**Supplementary Figure 4D**) (23). We next evaluated if MHC I deletion impacts CD8^+^ T cell function in both the spleen and liver. Isolated splenic or liver CD8^+^ T cells were cultured under unstimulated or stimulated conditions with CD3/CD28 to confirm functionality in the MHC I KO genotype. Using flow cytometry, we identified less splenic CD8^+^ T cells in the MHC I KO model and no differences between WT and Kb CD8^+^ T cells. Both MHC I KO and Kb CD8^+^ T cells responded to stimulation with increased activation (*CD44^+^, CD62L^-^*) and proliferation comparable to WT CD8^+^ T cells (**Supplementary Figures 4E–G**). Previous studies have evaluated the impact of Kb deletion in myeloid cells on CD8^+^ T cell development. These studies reported no significant changes in the proportion of CD8^+^ T cells or changes in the TCR repertoire diversity (18). Liver CD8^+^ T cells in the MHC I KO mice did not to respond to CD3/CD28 stimulus to the same extent as WT mice with less total CD8^+^ TCRβ^+^ cells present under stimulated conditions. However, MHC I KO CD8^+^ T cells were still able to response to stimulus with increased activation and proliferation (**Supplementary Figures 4H–J**).

To determine if altered MHC I expression impacts NK1.1+ and NK T cells, immune cells from livers of chow WT, MHC I KO, Kb, and LysM Kb KO mice were evaluated. Flow cytometry analysis revealed no significant differences in *NK1.1^+^TCRβ^-^* and *NK1.1^+^TCRβ^+^* cells between WT and MHC I KO, Kb, and LysM Kb KO mice (**Supplementary Figures 5A,B**). Additionally, no significant changes were detected in *NK1.1^+^TCRβ^+^* subsets for *CD4^+^* and *CD4^-^CD8^-^* subsets (**Supplementary Figures 5D, E**). Previous studies support the lack of NK response to the missing MHC I as studies in β2m KO mice are not autoreactive and are tolerant to the MHC I deficiency (24–26).

Taken together, our results show that the absence of H2Kb and H2Db, and myeloid specific deletion of H2Kb protects against NASH associated CD8^+^ T cell activation whereas H2Kb expression, in the absence of H2Db, increases CD8^+^ T cell activation.

### 3.3 MHC I KO protects against NASH associated inflammation and fibrosis while LysM Kb KO only protects against fibrosis

**Figure 3:**
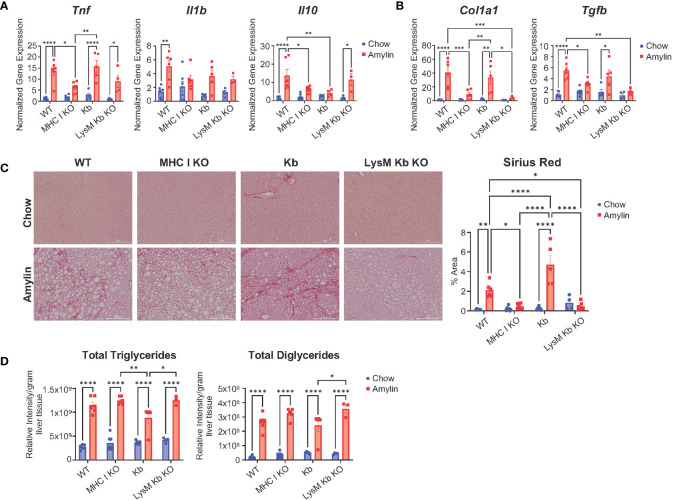
MHC I KO and LysM Kb KO are protective against NASH. WT, MHC I KO, Kb, or LysM Kb KO mice were on diet for 28 wks (n=3-7 per group, in two replicate cohorts). **(A, B)** Hepatic gene expression. **(C)** Representative figures of Sirius red staining of mouse liver sections and quantification. **(D)** Quantification of liver triglyceride and diglyceride lipid species detected by LC-MS/MS analysis. Data shown as the mean ± SEM. Two-way ANOVA were performed and determined significant by P<0.05 (*), P<0.01 (**), P<0.001 (***), and P<0.0001 (****).

We next evaluated the impact of MHC I function on hepatic inflammation and fibrosis. In the absence of H2Kb and H2Db, mice were protected from diet induced increases of inflammatory genes *Tnf*, *Il1b and Il10* and compared to WT amylin mice. However, Kb and LysM Kb KO mice demonstrated significant increases in *Tnf* with amylin diet. MHC I KO and Kb mice had significant reductions in Il10 expression while LysM Kb KO mice increased with amylin diet (**Figure 3A**). Interestingly, MHC I KO and LysM Kb KO mice were protected from diet induced fibrosis with significant reductions in hepatic gene expression of fibrotic markers *Col1a1* and *Tgfβ* in contrast to the significant increases in WT and Kb mice on amylin (**Figure 3B**). In correlation with gene expression, MHC I KO and LysM Kb KO mice showed significant reductions in Sirius red staining compared to WT and Kb amylin mice. Interestingly, Kb amylin mice demonstrated significantly increased collagen deposition compared to WT amylin mice. (**Figure 3C**). To examine hepatic steatosis, we performed mass spectrometry analysis of lipid species in the liver. We found total liver triglycerides and diglycerides were significantly upregulated with amylin diet and not impacted by genotype (**Figure 3D**).

Our findings suggest that H2Kb and H2Db are necessary for NASH induced CD8^+^ T cell activation, inflammation, and fibrosis but do not regulate hepatic lipid accumulation. Additionally, H2Kb expression in the absence of H2Db leads to advanced NASH fibrosis. Meanwhile, H2Kb specific knockout in myeloid cells reduced fibrosis but not NASH associated inflammation.

### 3.4 Ncf2 is a unique H2Kb restricted peptide in NASH

**Figure 4:**
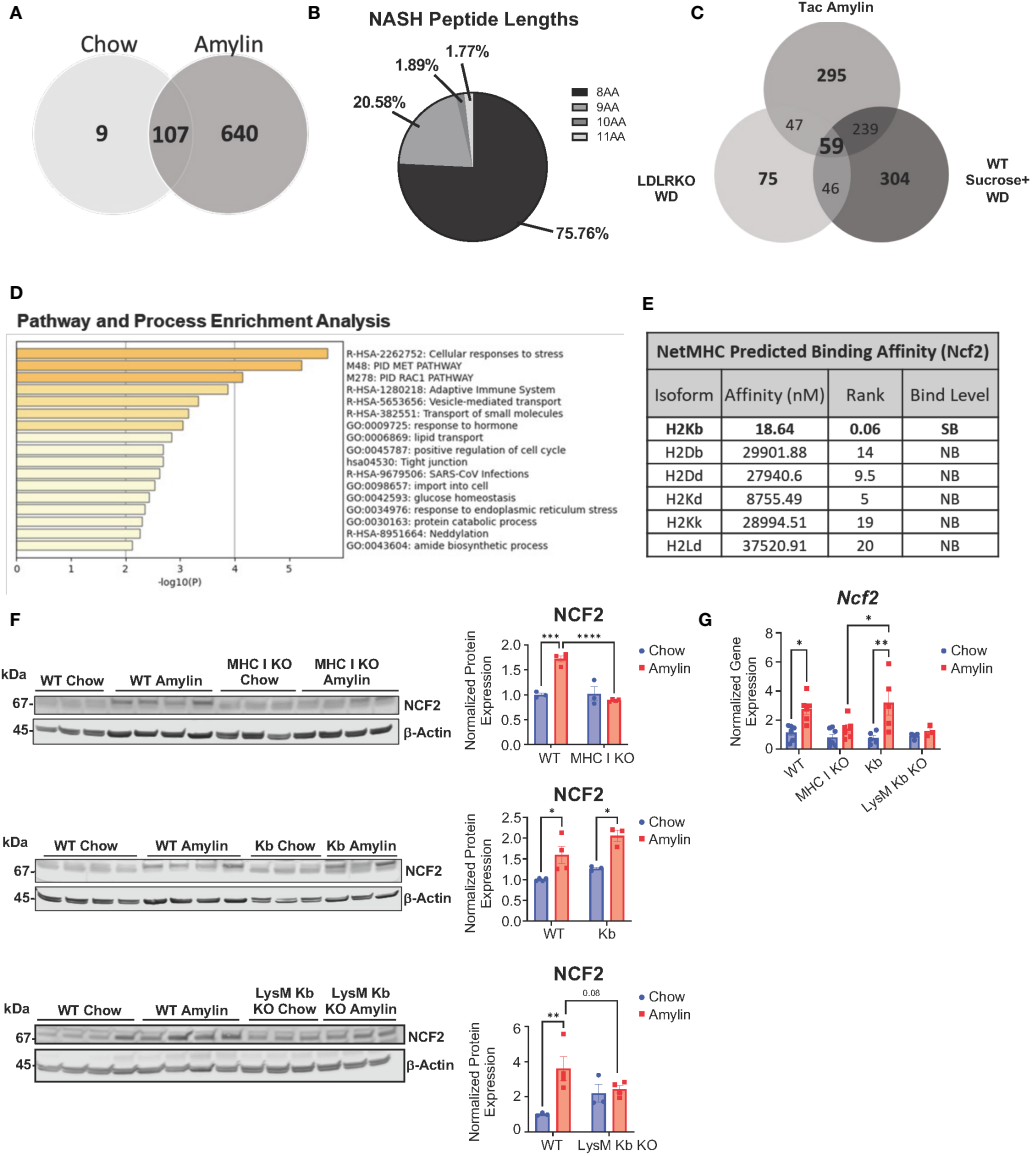
Unique H2Kb restricted peptides in NASH. Taconic mice on chow or amylin diet for 28 wks (n=5 mice per group, 2 replicate cohorts). LDLRKO mice on chow or WD for 12 wks (n=3-4 mice per group, 2 replicate cohorts). WT mice on chow or WD and sucrose water for 25 wks (n=5 mice per group, 1 cohort). WT, MHC I KO, Kb, or LysM Kb KO mice on chow or amylin diet (n= 3-7 mice per group, 2 replicate cohorts). **(A)** Venn diagram of the Taconic NASH model chow vs NASH peptides. **(B)** Pie chart of percentage of peptides with different amino acid lengths from Tac NASH model. **(C)** BioVenn filtered unique NASH peptides. **(D)** Bar graph of enriched terms across input gene lists, colored by p-values using Metascape. **(E)** NetMHC predicted binding affinities for Ncf2 peptide to MHC I isoforms for strong binders (SB) and non-binders (NB). Total liver Ncf2 protein **(F)** and gene expression **(G)** in WT, MHC I KO, Kb, and LysM Kb KO mice. Data shown as the mean ± SEM. Two-tailed unpaired Student’s t-tests were performed for two group comparisons. Two-way ANOVA was performed for multiple genotypes versus diet studies. Data was considered statistically significant for P<0.05 (*), P<0.01 (**), P<0.001 (***), and P<0.0001 (****).

Given H2Kb expression is important for CD8^+^ T cell activation, we aimed to identify H2Kb restricted peptides in NASH. Peptides were isolated from livers of NASH mice using immunoaffinity chromatography in three different mouse models of NASH (LDLRKO, Tac, and WT) compared to chow controls. Using BioVenn filtering analysis, we identified unique NASH peptides in NASH mouse models by removing chow associated peptides (**Figure 4A**). NASH peptides demonstrated a preference for 8 amino acids in length (**Figure 4B**, **Supplementary Figures 6A, B**). BioVenn filtering identified 59 NASH peptides found in all three mouse models of NASH (**Figure 4C**). Pathway analysis of peptide related proteins revealed enrichment in pathways such as cellular response to stress, adaptive immune system, response to endoplasmic reticulum stress, and protein catabolic processes (**Figure 4D**). Using NetMHCPan-4.1, all peptides were predicted to be strong H2Kb binders (**Supplementary Table 2**).

Interestingly, the peptide VHYKYTVV (Ncf2 peptide) was found in all NASH models and predicted to strongly bind to H2Kb with high specificity and not predicted to bind to any other MHC I isoforms (**Figure 4E**, **Supplementary Figure 6C**). The Ncf2 peptide is associated with the p67phox protein, a critical subunit for NADPH oxidase activity (27–29). Protein and gene expression of Ncf2 was significantly upregulated in the LDLRKO NASH model (**Supplementary Figures 6D, E**). Interestingly, MHC I KO and LysM Kb KO mice showed reduced Ncf2 protein and gene expression compared to WT amylin mice. Additionally, Kb amylin behaved similar to WT amylin mice with increased Ncf2 expression (**Figures 4F, G**).

### 3.5 Ncf2 peptide activates hepatic NASH CD8^+^ T cells *in vitro*

**Figure 5:**
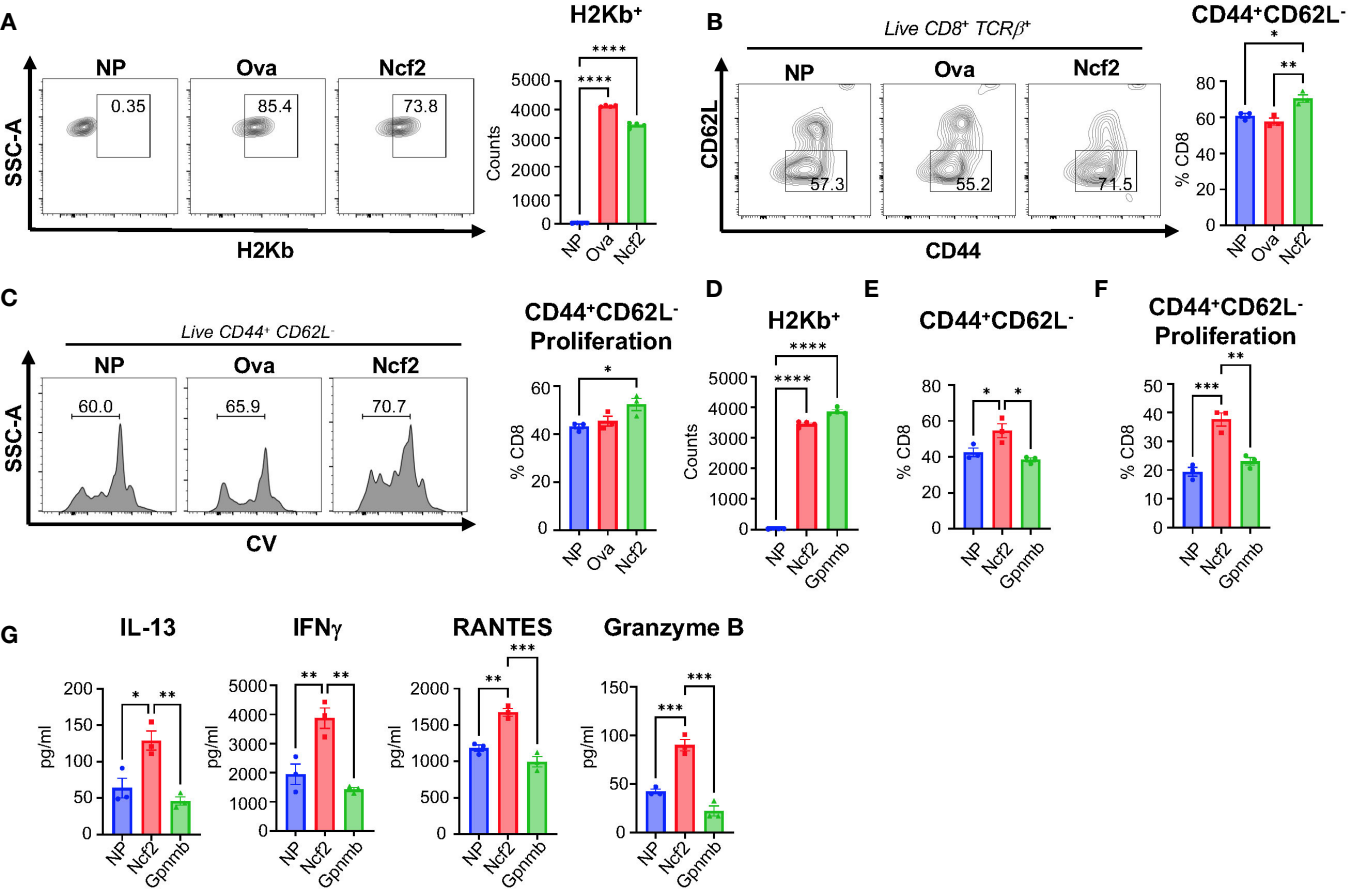
Ncf2 peptide activates hepatic NASH CD8^+^ T cells *in vitro*. Hepatic CD8^+^ T cells were isolated from LDLRKO NASH mice (2 combined NASH livers per cohort, 4 replicate cohorts, n=3 technical replicates per study). **(A)** Flow cytometry analysis of H2Kb expression to determine binding with NP, Ova, or Ncf2 peptide pulsed RMA-S cells (n=4 technical replicates, 3 replicate studies). T cell activation assay of hepatic CD8^+^ T cells co-cultured with NP, Ova, or Ncf2 peptide pulsed RMA-S cells. Cells were harvested on day 3 for flow cytometry analysis of CD8^+^ T cells (*CD8^+^TCRβ^+^*) and gated for **(B)** activation (*CD44^+^ CD62L^-^*) and **(C)** proliferation. **(D)** Representative flow cytometry analysis of peptide binding assay for NP, Ncf2, and Gpnmb peptides (n=4, 3 replicate studies). **(E, F)** Day 5 harvested cells for flow cytometry analysis of CD8^+^ T cell activation **(E)** and proliferation **(F)**. **(G)** Cytokine analysis of media from day 5 of the T cell activation assay with hepatic LDLRKO NASH CD8^+^ T cells. Flow plots show percentage of parent gate. Data shown as the mean ± SEM. Two-tailed unpaired Student’s t-tests was performed for data sets with 2 groups and Two-way ANOVA was performed for groups more than 2 and was considered statistically significant for P<0.05 (*), P<0.01 (**), P<0.001 (***), and P<0.0001 (****).

We determined the ability of the Ncf2 peptide to activate NASH CD8^+^ T cells *in vitro*. To confirm Ncf2 predicted H2Kb binding, a peptide binding assay was performed using RMA-S cells. Cells were pulsed with a vehicle control containing no peptide (NP), a known H2Kb binding peptide ovalbumin (Ova), or the Ncf2 peptide. Flow cytometry analysis confirmed the peptides Ncf2 and Ova bind to H2Kb compared to NP control (**Figure 5A**). Next, we investigated whether Ncf2 could activate hepatic CD8^+^ T cells isolated from NASH mice. Using the LDLRKO NASH model, hepatic CD8^+^ T cells were isolated and co-cultured with RMA-S cells pulsed with NP, Ova, or Ncf2 and harvested for flow analysis at days 3 and 5. By day 3 samples treated with the Ncf2 peptide showed increased CD8^+^ T cell activation (*CD44^+^ CD62L^-^*) and proliferation compared to both NP and Ova treated cells (**Figures 5B, C**). Additionally, the peptide binding assay confirmed peptide binding of another candidate peptide Gpnmb found in the LDLRKO NASH model (**Figure 5D**). To test whether Gpnmb activates CD8^+^ T cells, RMA-S cells were treated with NP, Ncf2, or Gpnmb peptides and co-cultured with hepatic CD8^+^ T cells. Interestingly, by day 5 only Ncf2 showed significant increases in both CD8^+^ T cell activation and proliferation compared to both NP control and Gpnmb treated cells (**Figures 5E, F**). To test for cytotoxicity, cytokines from the media were evaluated and showed significant increases in IL-13, IFNγ, RANTES, and GRANZYME B by day 5 in Ncf2 treated cells compared to NP and Gpnmb (**Figure 5G**).

Comparable to the LDLRKO NASH model, hepatic CD8^+^ T cells from the Tac NASH model also demonstrated significant increases in CD8^+^ T cell activation and proliferation when exposed to the Ncf2 peptide compared to NP control by day 3 (**Supplementary Figures 7A, B**). Splenic CD8^+^ T cells also demonstrated significant increases in T cell activation and proliferation in response to the Ncf2 peptide under both stimulated (**Supplementary Figures 7C, D**) and unstimulated conditions supplemented with IL-2 (**Supplementary Figures 6E, F**) in the Tac NASH model by day 5. Additionally, WT NASH and LDLRKO NASH models demonstrated CD8^+^ T cell activation and proliferation by day 5 (**Supplementary Figures 7C–H**). Splenic CD8^+^ T cells activated by the Ncf2 peptide shared a similar cytokine profile to hepatic CD8^+^ T cells with significant increases in IFNγ, RANTES, and GRANZYME B (**Supplementary Figure 7K**).

**Figure 6:**
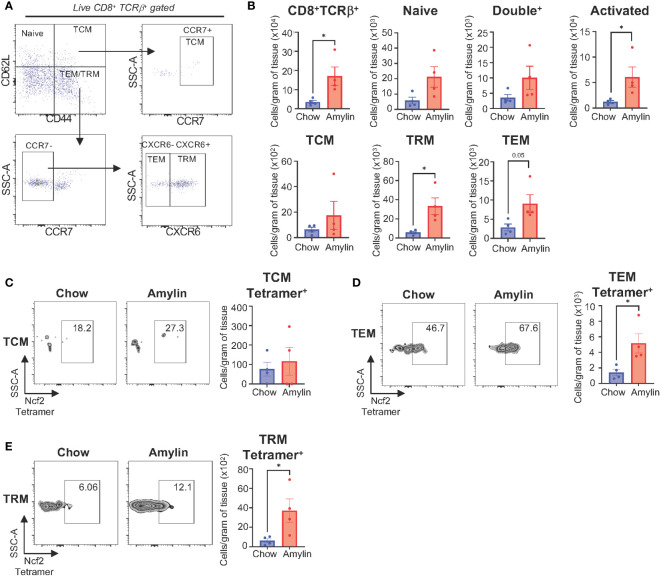
Ncf2 reactive CD8^+^ T cells are detected *in vivo*. Tac NASH mice were fed chow or amylin diet for 28 wks (n=4 per group). **(A)** Flow cytometry CD8^+^ T cell subsets gating strategy from CD8^+^Tcrβ^+^ liver lymphocytes. **(B)** Quantification of flow cytometry data. Representative Ncf2 Tetramer positive flow cytometry plots and quantification from liver CD8^+^ T cell subsets: **(C)** TCM, **(D)** TEM, and **(E)** TRM cells. Quantification of flow cytometry data. Flow plots show percentage of parent gate. Data shown as the mean ± SEM. Two-tailed unpaired Student’s t-tests was performed and considered statistically significant for P<0.05 (*).

To confirm CD8^+^ T cell reactivity to the Ncf2 peptide, an H2Kb-Ncf2 tetramer was used to detect the presence of Ncf2 specific CD8^+^ T cells *in vivo*. Immune cells were isolated from livers of Taconic chow or Amylin mice and stained for flow cytometry. Flow analysis confirmed increased CD8^+^ T cells and activation in NASH. Additionally, CD8^+^ T cell subsets were evaluated and identified significant increases in T cell effector memory (TEM, *CD62L^-^, CD44^+^, CCR7^-^, CXCR6^-^*) and T cell resident memory subsets (TRM, *CD62L^-^, CD44^+^, CCR7^-^, CXCR6^+^*) with no changes in T cell central memory cells (TCM, *CD6L2+, CD44^+^, CCR7^+^*) (**Figures 6A, B**). TEM and TRM CD8^+^ T cell subsets showed a significant increase in Ncf2 Tetramer signal compared to chow controls with no significant changes detected in TCMs (**Figures 6C–E**). These results demonstrate that H2Kb restricted peptide Ncf2 can activate both hepatic and splenic CD8^+^ T cells from NASH mice *in vitro* and with increased activation, proliferation, and cytotoxicity. Additionally, NASH mice have increased Ncf2 reactive hepatic TEM and TRM CD8^+^ T cells detected *in vivo*.

### 3.6 Ncf2 peptide is not present during fibrosis resolution

**Figure 7:**
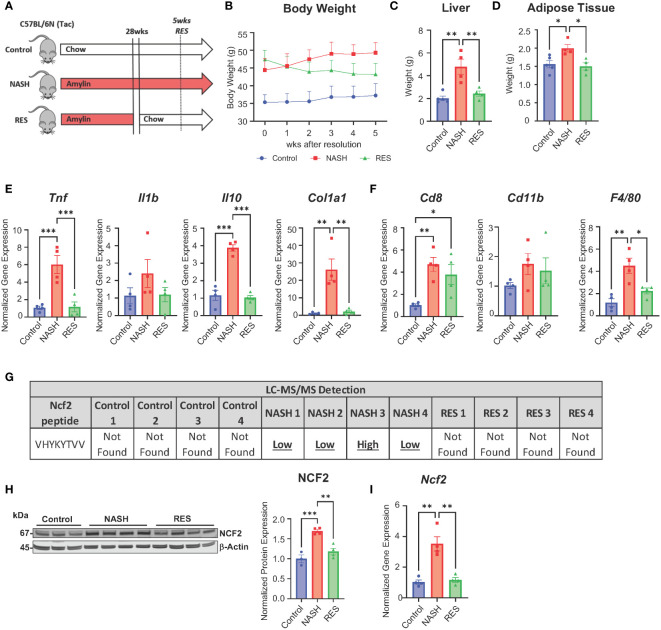
Ncf2 peptide is not found during NASH resolution. Tac NASH mice were fed chow or amylin diet for 33 wks. Tac RES mice were fed amylin diet for 28 wks and then switched to chow diet for 5 wks (n=4, 2 replicate cohorts). **(A)** Resolution study schematic. **(B)** Body weight progression. **(C)** Total liver weight. **(D)** Epididymal adipose tissue weight. **(E, F)** Hepatic gene expression. **(G)** LC-MS/MS detection of Ncf2 peptide from total livers of individual mice from control (chow), NASH, or RES (n=4). Total liver Ncf2 protein **(H)** and gene **(I)** expression. Data shown as the mean ± SEM. Two-way ANOVA was performed and considered statistically significant for P<0.05 (*), P<0.01 (**), and P<0.001 (***).

A recent study has shown that tissue-resident memory (TRM) CD8^+^ T cells play a key role in resolving fibrosis during resolution (RES). NASH RES can be established within 5 weeks of switching from a high fat high cholesterol diet to a chow diet. At 5 weeks of resolution CD8^+^ T cells remain elevated, but mice show reductions in inflammation and fibrosis (9). Utilizing this model concept, we investigated if the expression of peptide Ncf2 is altered during RES at 5 weeks when CD8^+^ T cells are still elevated (**Figure 7A**). As seen in previous studies, RES mice had significantly reduced body weight, liver weight, and adipose tissue compared to NASH mice after 5 weeks of resolution (**Figures 7B–D**) (9). Hepatic gene expression in RES mice showed significant reductions in inflammation (*Tnf* and *Il10)* and fibrosis (*Col1a1)* compared to NASH mice (**Figure 7E**). Additionally, gene expression for immune cell markers *Cd8* and *Cd11b* remained elevated during resolution whereas *F4/80* expression was significantly reduced compared to NASH mice (**Figure 7F**). Next, we used mass spectrometry to evaluate if the Ncf2 peptide is present during resolution. Interestingly, the Ncf2 peptide was only present under NASH conditions (**Figure 7G**). Ncf2 hepatic protein and gene expression was also reduced during resolution compared to NASH mice (**Figures 7H, I**). These results indicate that the Ncf2 peptide may be necessary for *in vivo* pathogenic CD8^+^ T cell activation in NASH and the presence of the peptide is dependent on dietary signals.

## 4 Discussion

In the present study, we demonstrate that hepatic CD8^+^ T cell activation in NASH is dependent on H2Kb driving liver fibrosis with no impact on hepatic steatosis. H2Kb only expressing mice displayed worsened fibrosis in the absence of H2Db suggesting that H2Kb is the dominant isoform driving disease progression and H2Db may be protective in NASH. Additionally, myeloid cell expression of H2Kb is important for regulating CD8^+^ T cell activation and fibrosis in NASH. NASH mice also demonstrate a unique H2Kb immunopeptidome and functional characterization of the Ncf2 peptide demonstrated the peptide induced activation, proliferation, and cytokine secretion of NASH CD8^+^ T cells. Under fibrosis resolution the Ncf2 peptide is not detected, suggesting an antigen unique to the NASH environment. We determined that both gene and protein expression of Ncf2 were elevated during NASH and dependent on MHC class I expression. *Ncf2* gene expression is also upregulated in other liver fibrosis rodent models (30–32). This is surprising as peptides are normally generated during proteasomal degradation leading to reduced protein levels. This may be due to other cell types regulating p67phox expression versus a specific APC cell undergoing degradation or presenting the Ncf2 peptide. It remains to be determined the origin of this Ncf2 peptide and how MHC class I molecules regulate specific subsets of CD8^+^ T cells during NASH development and progression.

Our data demonstrates that MHC I deficiency and H2Kb deficiency in myeloid cells can protect against liver fibrosis and CD8^+^ T cell activation in NASH. In contrast, a recent study argues that CD8^+^ T cells are activated through B cell stimulation through the IgA-FcR signaling in a choline deficient high fat (CD-HFD) model (33). This study showed that *in vitro* blocking of MHC I with an MHC I antibody is ineffective at preventing NASH CD8^+^ T cell activation by intestinal B cells (33). However, previous work has shown that in a methionine and choline deficient high fat diet (MCD) model of NASH, CD8^+^ T cells did not regulate hepatic inflammation, fibrosis, or stellate cell activation (7). Alternatively, β2m KO studies have shown that when using the CD-HFD model mice were protected from CD8^+^ T cell activation, inflammation, and fibrosis. However, this model does not account for just MHC I antigen presentation as β2m is also present on CD1, Qa-1, and neonatal Fc receptor (FcRn) receptors impacting more than just CD8^+^ T cells (5). Differences in choline content of the NASH diets may contribute to the factors that regulate CD8^+^ T cell activation and function. CD8^+^ T cells function may be altered under choline deficient conditions as activation of the T cell receptor (TCR) through antigen presentation upregulates components of their cholinergic system (34). Likewise, Tap1 deficient mice are protected from fructose induced NASH in mice (35). Therefore, targeting MHC I with an antibody may be ineffective as these molecules are typically bound with peptides when presented at the cell surface which could prevent the binding of blocking antibodies. Further studies are necessary to clarify the mechanisms of CD8^+^ T cell activation across various dietary models of NASH. Alternative methods are also needed to better target MHC I molecules bound to specific NASH peptides.

Comparable to our findings, in a mouse model of cerebral malaria deficiency of H2Kb and H2Db led to reductions in CD8^+^ T cell activation resulting in improved survival (36). Using the Kb and LysM Kb KO mouse models our data demonstrates that H2Kb on myeloid cells is required for regulating CD8^+^ T cell activation and fibrosis in NASH. Interestingly, studies are beginning to highlight the distinct roles of H2Kb and H2Db in priming CD8^+^ T cells and disease progression (37). In the context of Theiler’s murine encephalomyelitis virus (TMEV) infection, H2Db, but not H2Kb, controls the development of brain atrophy (38, 39). In addition, CD8^+^ T cell activation through an H2Db restricted TMEV-derived peptide contributes to brain atrophy (39). In lymphocytic choriomeningitis virus (LCMV) mouse models, deficiency of H2Db led to more severe liver pathology, increased hepatocyte apoptosis, and increased H2Kb restricted cytotoxic CD8^+^ T cell numbers compared to WT and H2Kb KO mice (40). These studies in conjunction with our data, suggest that antigen presentation by different MHC I isoforms is important in regulating the type of CD8^+^ T cell responses and are important factors in infection and chronic disease progression.

Hepatic inflammation has been linked to bone marrow derived monocyte infiltration into the liver driving NAFLD progression. Studies have shown that myeloid cells in the liver are also key players involved in fibrosis development (41–44). A recent transcriptomic study characterized macrophage subsets in NASH identifying increased pro-fibrotic and M2 macrophage subsets significantly correlated with degree of fibrosis in human patients (45). Our studies identified that myeloid cells have increased H2Kb expression demonstrating a potential mechanism by which antigens are presented to CD8^+^ T cells in NASH driving fibrosis. This is further supported by H2Kb deletion on myeloid cells protecting mice against NASH induced CD8^+^ T cell activation and fibrosis. CD8^+^ T cells are also known to interact with hepatocytes (14), hepatic stellate cells, in addition to infiltrating myeloid cells (7) via antigen presentation. In particular, the p67phox protein associated with the Ncf2 peptide we identified is expressed by monocytes, macrophages, hepatocytes, and dendritic cells under NASH conditions. Cross presentation by dendritic cells may also provide a mechanism for antigen presentation. Upon examination of dendritic subsets, classical dendritic cells (cDC1) were found to be elevated and drive liver injury and CD8^+^ T cell activation (46). Similarly, adipose tissue dendritic cells are linked to metabolic dysfunction, were depletion of conventional dendritic cells prevents HFD model induced inflammation (47). Additionally, B cells have been highlighted to contribute to the progression of NASH through the production of pro-inflammatory mediators and antigen presentation. Studies have shown that B cell deficiency protects mice against liver fibrosis and inflammation in diet induced NASH models (48, 49). Studies also suggest that the gut microbiome can promote NASH progression through activation of intrahepatic B cells through microbial factors (49). However, MHC II is often upregulated in patients with NASH preceding CD8^+^ T cell infiltration into the liver (48). It is known that B cells can activate CD8^+^ T cells though IgA-FcR signaling, however it remains to be determined how B cell MHC I expression changes during NASH and how this impacts CD8^+^ T cell activation (33). These findings highlight the ability of alternative APCs for antigen presentation that have not been evaluated in NASH.

During NASH progression and fibrosis resolution, analysis of the TCR repertoires of hepatic memory CD8^+^ T cells demonstrated less diversity indicating possible antigen regulation (9). In high fat fed mice, we discovered previously that hepatic CD8^+^ T cells share distinct clonotypes in their TCR repertoires with CD8^+^ T cells from adipose tissue (50). In the current study we examined the hepatic immunopeptidome, which consists of all antigens bound to H2Kb in normal and NASH mouse livers. We identified 59 unique H2Kb dependent antigens in NASH livers with peptides associated to pathways such as tight junctions and cellular stress. Of these NASH peptides, we identified a CD8^+^ T cell reactive Ncf2 peptide. This peptide is associated with the p67phox cytosolic subunit that makes up part of the NOX2 NADPH oxidase protein complex (29, 51). Previous studies have shown that NOX2 is increased in NASH and NOX2 deficient mice are protected from diet induced steatosis and insulin resistance (52). Interestingly, in a lupus mouse model, knockdown of just the p67phox subunit reduces splenic CD8^+^ T cells suggesting the Ncf2 peptide could be important for also activating CD8^+^ T cells in other inflammatory diseases (53). Targeting the Ncf2 peptide or protein could provide new alternative therapies for targeting CD8^+^ T cell activation in NASH and other inflammatory diseases.

The importance of myeloid specific H2Kb antigen presentation for CD8^+^ T cell activation in NASH was highlighted in these studies. However, it remains to be determined the role of other candidate APC types and the origin of the Ncf2 peptide. The role of antigen presentation by other APCs such as hepatocytes, dendritic cells, and B cells can be evaluated using the Kb LoxP mouse model. Additionally, an effort to determine the source of the Ncf2 peptide needs to be addressed. It is unknown if the Ncf2 peptide is unique to the liver or generated in other metabolic tissues such as the intestine or adipose tissue. Gut microbes and oxidative stress related antigens may be generated in the intestine and travel to the liver driving CD8^+^ T cells. It also remains to be determined if the Ncf2 peptide and Ncf2 reactive CD8^+^ T cells are present in NASH in humans. Future studies targeting the Ncf2 protein or peptide may serve as a promising therapeutic target for regulating fibrosis in NASH.

## Data availability statement

The data presented in the study can be found in the MassIVE Repository, accession number PXD047295. 

## Ethics statement

The animal study was approved by Institutional Animal Care and Use Committee (IACUC) at North Carolina State University. The study was conducted in accordance with the local legislation and institutional requirements.

## Author contributions

VA: Data curation, Formal analysis, Methodology, Visualization, Writing – original draft, Writing – review & editing. LC: Data curation, Formal analysis, Methodology, Writing – review & editing. TW: Methodology, Writing – review & editing. JH: Resources, Writing – review & editing. PH: Resources, Writing – review & editing. HA: Formal analysis, Writing – review & editing. GS: Data curation, Writing – review & editing. XL: Data curation, Methodology, Writing – review & editing. ML: Data curation, Writing – review & editing. AJ: Methodology, Resources, Writing – review & editing. AK: Conceptualization, Data curation, Formal analysis, Funding acquisition, Investigation, Methodology, Project administration, Resources, Supervision, Validation, Writing – original draft, Writing – review & editing.
