# Supplementary material for: Myeloid cell MHC I expression drives CD8+ T cell activation in nonalcoholic steatohepatitis
Source: Front Immunol. 2024 Jan 11;14:1302006. doi: 10.3389/fimmu.2023.1302006 (PMC10808415; doi:10.3389/fimmu.2023.1302006)
Supplement: Supplementary file 1 [file DataSheet_1.pdf]

Figure S1

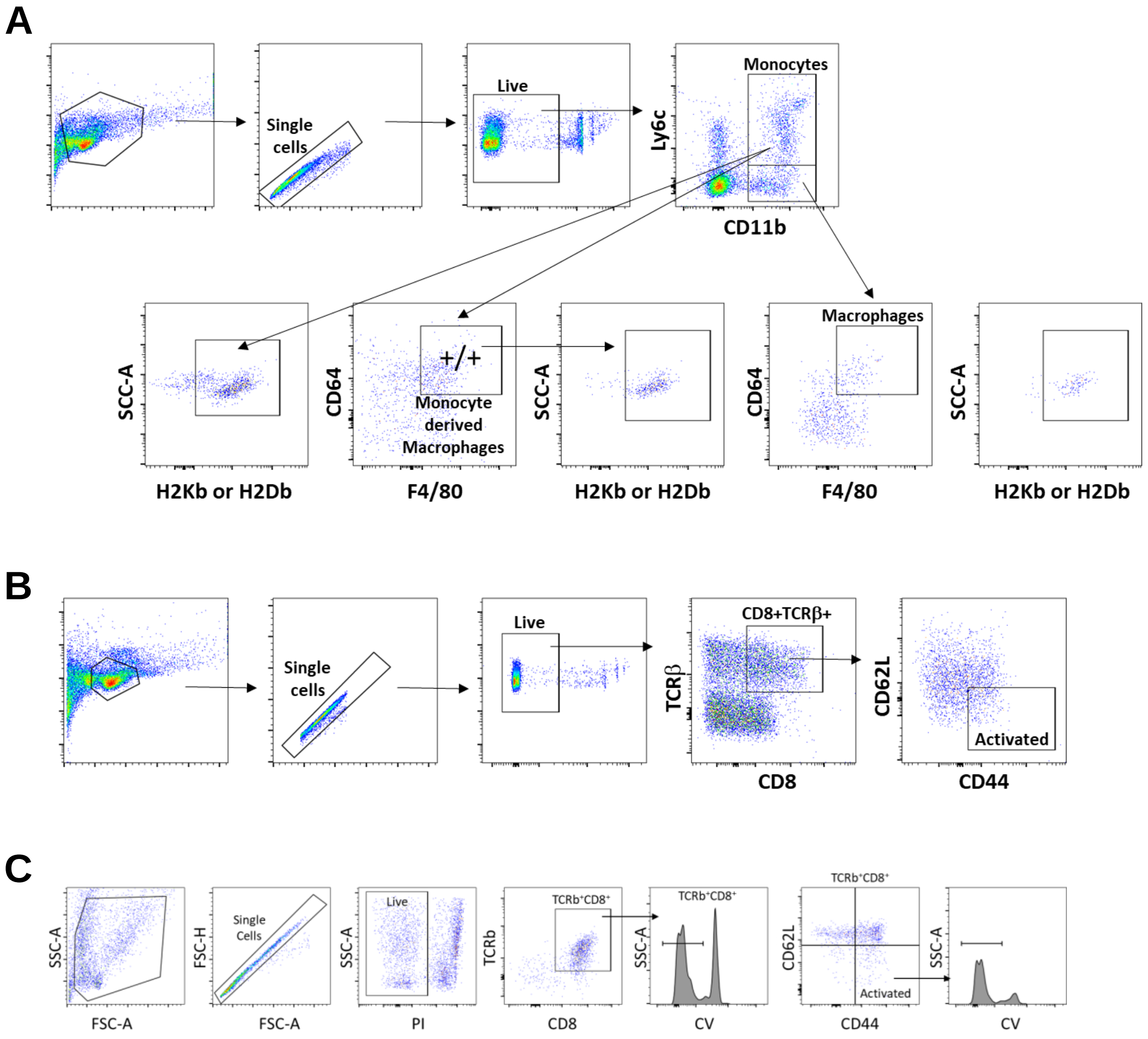
A
Single cells
Live
Monocytes
Ly6c
CD11b
SCC-A
H2Kb or H2Db
CD64
+/+
Monocyte derived Macrophages
F4/80
SCC-A
H2Kb or H2Db
Macrophages
CD64
F4/80
SCC-A
H2Kb or H2Db
B
Single cells
Live
CD8+TCRβ+
TCRβ
CD8
CD62L
Activated
CD44
C
SSC-A
FSC-A
Single Cells
FSC-H
FSC-A
Live
SSC-A
PI
TCRb⁺CD8⁺
TCRb
CD8
TCRb⁺CD8⁺
SSC-A
CV
TCRb⁺CD8⁺
CD62L
Activated
CD44
SSC-A
CV

Figure. S1. Flow cytometry gating strategies. (A) Myeloid cell flow gating strategies. **(B)** T cell flow gating strategies. **(C)** T cell flow gating strategies for T cell activation assays.

Figure S2

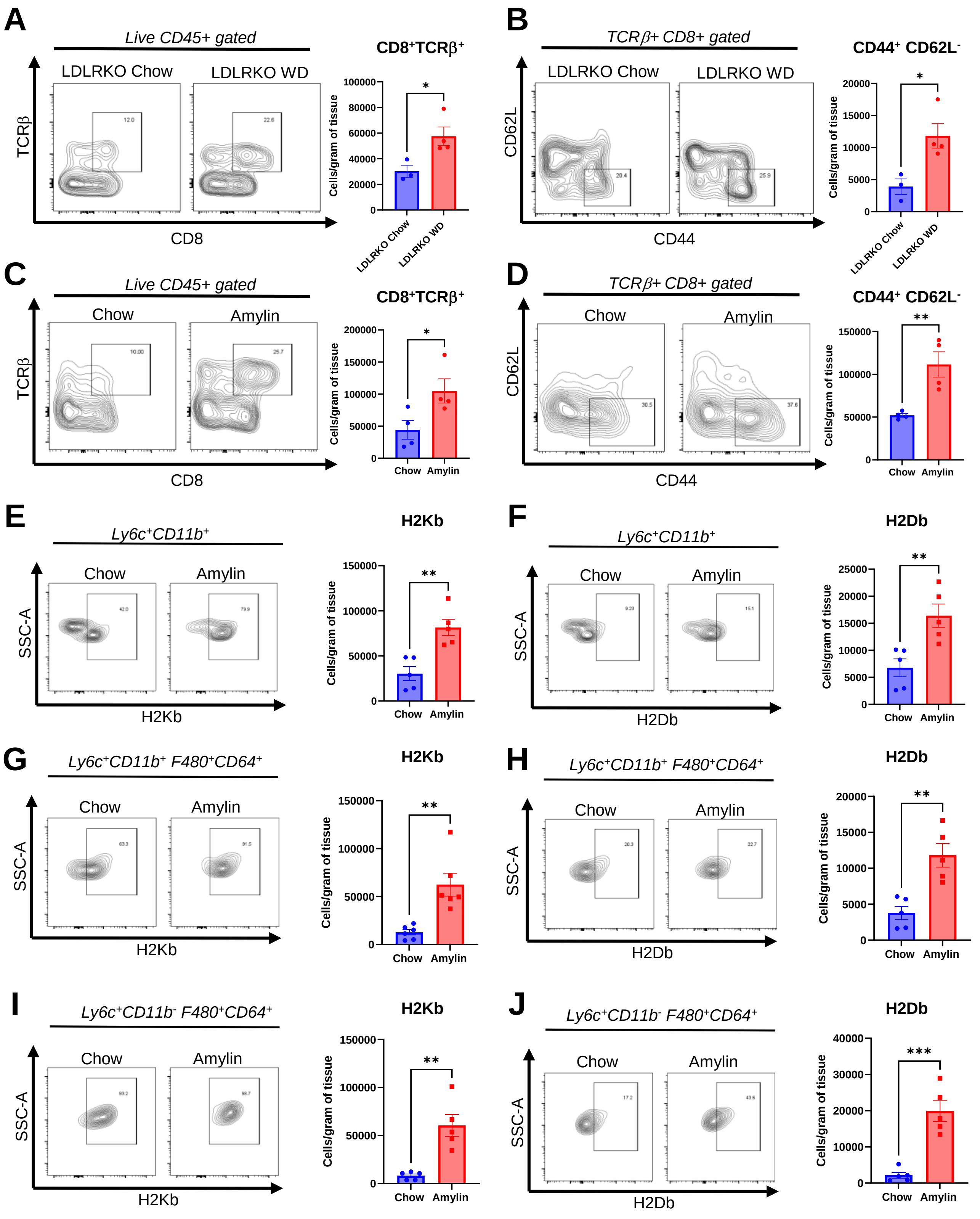
A
Live CD45+ gated
LDLRKO Chow
LDLRKO WD
TCRβ
CD8
CD8⁺TCRβ⁺
Cells/gram of tissue
B
TCRβ+ CD8+ gated
CD62L
CD44
CD44⁺ CD62L⁻
C
Chow
Amylin
D
E
Ly6c⁺CD11b⁺
SSC-A
H2Kb
F
H2Db
G
Ly6c⁺CD11b⁺ F480⁺CD64⁺
H
I
Ly6c⁺CD11b⁻ F480⁺CD64⁺
J

Figure. S2. $CD8^+$ T cells are upregulated in NASH with increased H2Kb and H2Db in myeloid cells. LDLRKO mice on chow or WD for 12 wks (n=3-4 mice per group, 2 replicate studies) (A-B). Taconic mice on chow or amylin diet for 28 wks (n=5 mice per group, 2 replicate studies) (C-D). WT amylin mice on diet for 28 wks (n=4, 2 replicate studies) (E-J). Flow analysis of $CD8^+$ $TCRb^+$ cells **(A,C)** and activation ($CD44^+CD62L^-$) **(B,D).** Representative flow plots of monocyte H2Kb **(E)** and H2Db **(F)** expression. Representative flow plots of monocyte derived macrophage H2Kb **(G)** and H2Db **(H)** expression. Representative flow plots of macrophage H2Kb **(I)** and H2Db **(J)** expression. Data shown as the mean ± SEM. Two-tailed unpaired Student's t-tests was performed and considered statistically significant for $P<0.05$ (*), $P<0.01$ (**), and $P<0.001$(***).

Figure S3

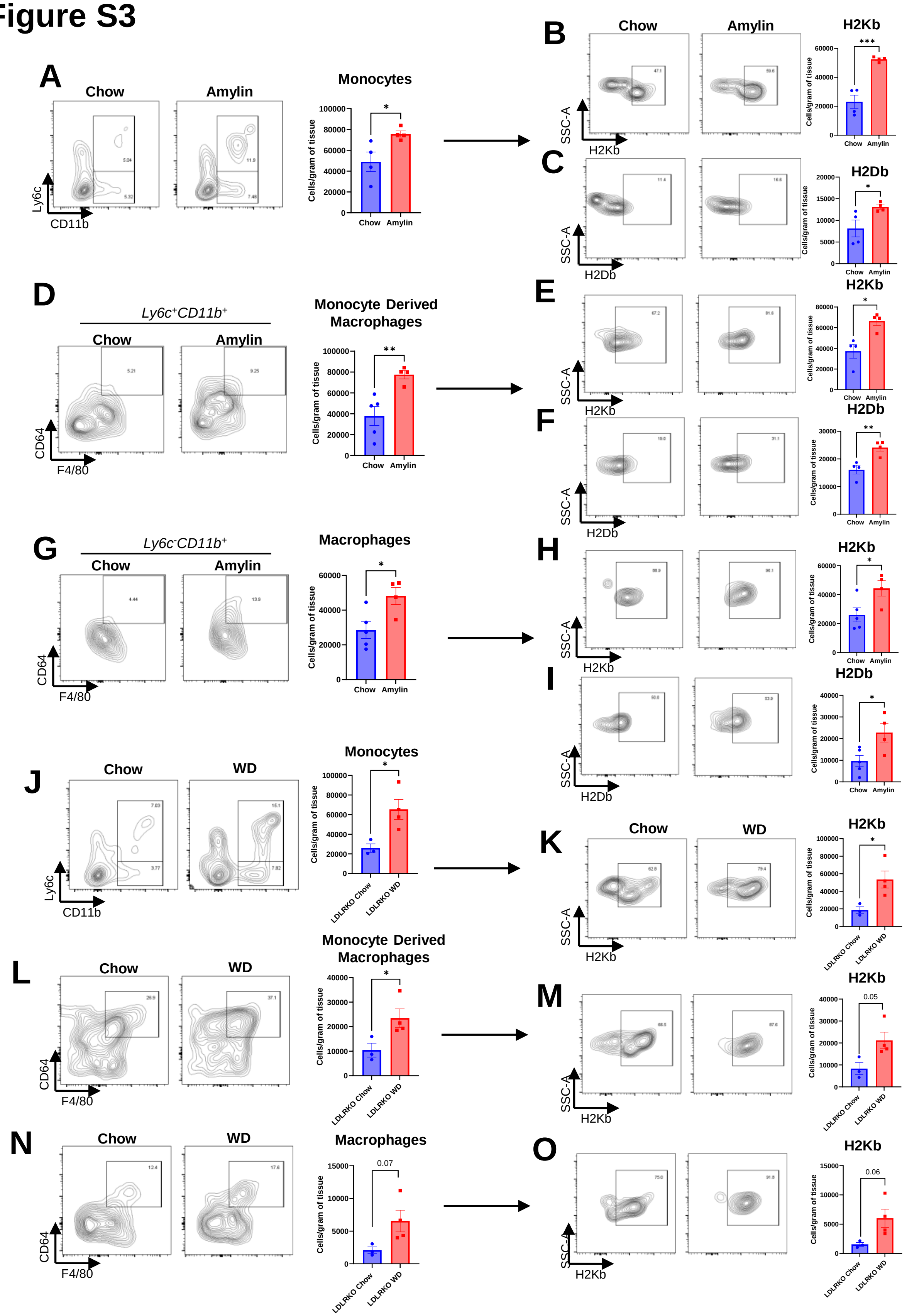

A
Chow
Amylin
Ly6c
CD11b
Monocytes
Cells/gram of tissue
B
C
D
Ly6c⁺CD11b⁺
CD64
F4/80
Monocyte Derived Macrophages
E
F
G
Ly6c⁻CD11b⁺
Macrophages
H
I
SSC-A
H2Kb
H2Db
J
WD
LDLRKO Chow
LDLRKO WD
K
L
M
N
O
0.05
0.07
0.06

Figure. S3. H2Kb and H2Db expression is upregulated in NASH myeloid cells. Taconic mice on chow or amylin diet for 28 wks (n=4 mice per group, 2 replicate cohorts) **(A-I)**. LDLRKO mice on chow or WD for 12 wks (n = 3-4 mice per group, 1 cohort) **(J-O)**. Taconic model flow analysis of monocytes ($Ly6c^{+}CD11b^{+}$) **(A),** monocyte H2Kb expression **(B)**, and monocyte H2Db expression **(C)**. Flow analysis of monocyte derived macrophages ($Ly6c^{+}CD11b^{+}CD64^{+}F480^{+}$) **(D),** monocyte derived macrophage H2Kb expression **(E)**, and monocyte derived macrophage H2Db expression **(F)**. Flow analysis of macrophages ($Ly6c^{-}CD11b^{+}CD64^{+}F480^{+}$) **(G),** macrophages H2Kb expression **(H)**, and macrophages H2Db expression **(I)**. LDLRKO model flow analysis of monocytes ($Ly6c^{+}CD11b^{+}$) **(J)** and monocyte H2Kb expression **(K)**. Flow analysis of monocyte derived macrophages ($Ly6c^{+}CD11b^{+}CD64^{+}F480^{+}$) **(L)** and monocyte derived macrophage H2Kb expression **(M)**. Flow analysis of macrophages ($Ly6c^{-}CD11b^{+}CD64^{+}F480^{+}$) **(N)** and macrophages H2Kb expression **(O).** Data shown as the mean ± SEM. Two-tailed unpaired Student's t-tests was performed and considered statistically significant for P<0.05 (*), P<0.01 (**), and P<0.001(***).

Figure S4

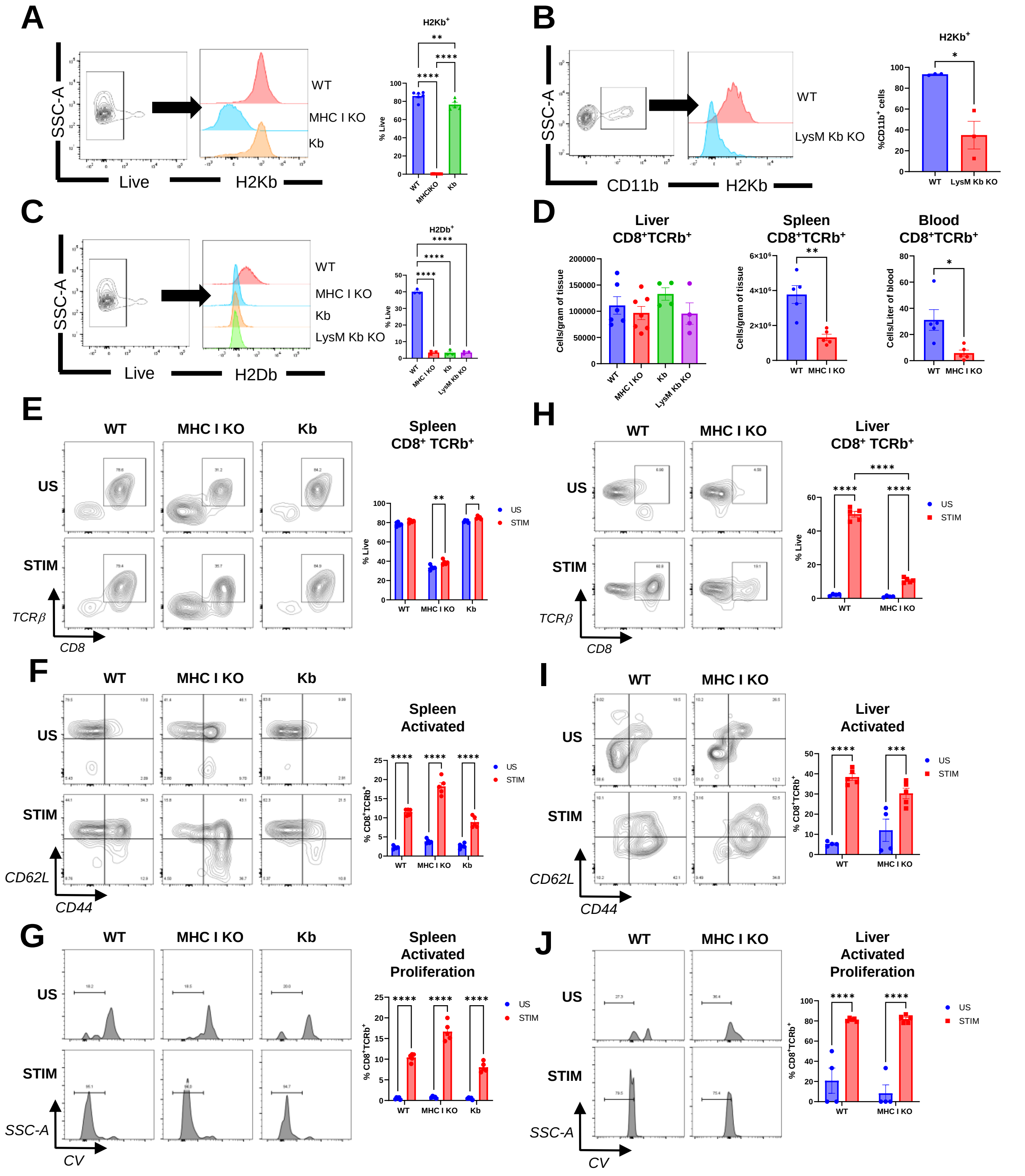

A
SSC-A
Live
H2Kb
WT
MHC I KO
Kb
H2Kb+
% Live
WT
MHCIKO
Kb
**
****
****
B
SSC-A
CD11b
H2Kb
WT
LysM Kb KO
H2Kb+
%CD11b+ cells
*
WT
LysM Kb KO
C
SSC-A
Live
H2Db
WT
MHC I KO
Kb
LysM Kb KO
H2Db+
% Live
****
WT
MHC I KO
Kb
LysM Kb KO
D
Liver
CD8+TCRb+
Cells/gram of tissue
WT
MHC I KO
Kb
LysM Kb KO
Spleen
CD8+TCRb+
Cells/gram of tissue
**
WT
MHC I KO
Blood
CD8+TCRb+
Cells/Liter of blood
*
WT
MHC I KO
E
WT
MHC I KO
Kb
US
STIM
TCRβ
CD8
Spleen
CD8+ TCRb+
% Live
**
*
US
STIM
WT
MHC I KO
Kb
H
WT
MHC I KO
US
STIM
TCRβ
CD8
Liver
CD8+ TCRb+
% Live
****
US
STIM
WT
MHC I KO
F
WT
MHC I KO
Kb
US
STIM
CD62L
CD44
Spleen
Activated
% CD8+TCRb+
****
US
STIM
WT
MHC I KO
Kb
I
WT
MHC I KO
US
STIM
CD62L
CD44
Liver
Activated
% CD8+TCRb+
****
***
US
STIM
WT
MHC I KO
G
WT
MHC I KO
Kb
US
STIM
SSC-A
CV
Spleen
Activated
Proliferation
% CD8+TCRb+
****
US
STIM
WT
MHC I KO
Kb
J
WT
MHC I KO
US
STIM
SSC-A
CV
Liver
Activated
Proliferation
% CD8+TCRb+
****
US
STIM
WT
MHC I KO

Figure. S4. Efficient H2Kb and H2Db knockout in MHC I KO, Kb transgenic mice, and Kb KO in myeloid cells with functional spleen $CD8^+$ T cells. WT, MHC I KO, Kb, LysM Kb KO chow mice (n=5-7 per group, three cohorts). Representative flow plots of isolated liver cells gated for: **(A)** total $H2Kb^+$ cells, **(B)** $CD11b^+$ cells $H2Kb^+$ expression, and **(C)** total $H2Db^+$ cells. **(D)** Flow cytometry data for $CD8^+TCRb^+$ cells in WT, MHC I KO, Kb, and LysM Kb KO chow mouse livers. T cell activation assay with sorted $CD8^+$ splenic T cells under stimulated and unstimulated conditions from WT, MHC I KO, and Kb mice harvested after 3 days (n=5, in triplicate). Flow cytometry analysis of $CD8^+TCRb^+$ cells **(E),** activation ($CD44^+$, $CD62L^-$) **(F),** and proliferation **(G).** T cell activation assay with sorted $CD8^+$ Liver T cells under stimulated and unstimulated conditions from WT or MHC I KO chow mice harvested after 3 days (n=4-5). Flow cytometry analysis of $CD8^+TCRb^+$ cells **(H),** activation ($CD44^+$, $CD62L^-$) **(I),** and proliferation **(J).** Data shown as the mean ± SEM. Two-tailed unpaired Student's t-tests for data with two groups and Two-way ANOVA was performed for more than two and was considered statistically significant for $P<0.05$ (*), $P<0.01$ (**), $P<0.001$(***), and $P<0.0001$(****).

Figure S5

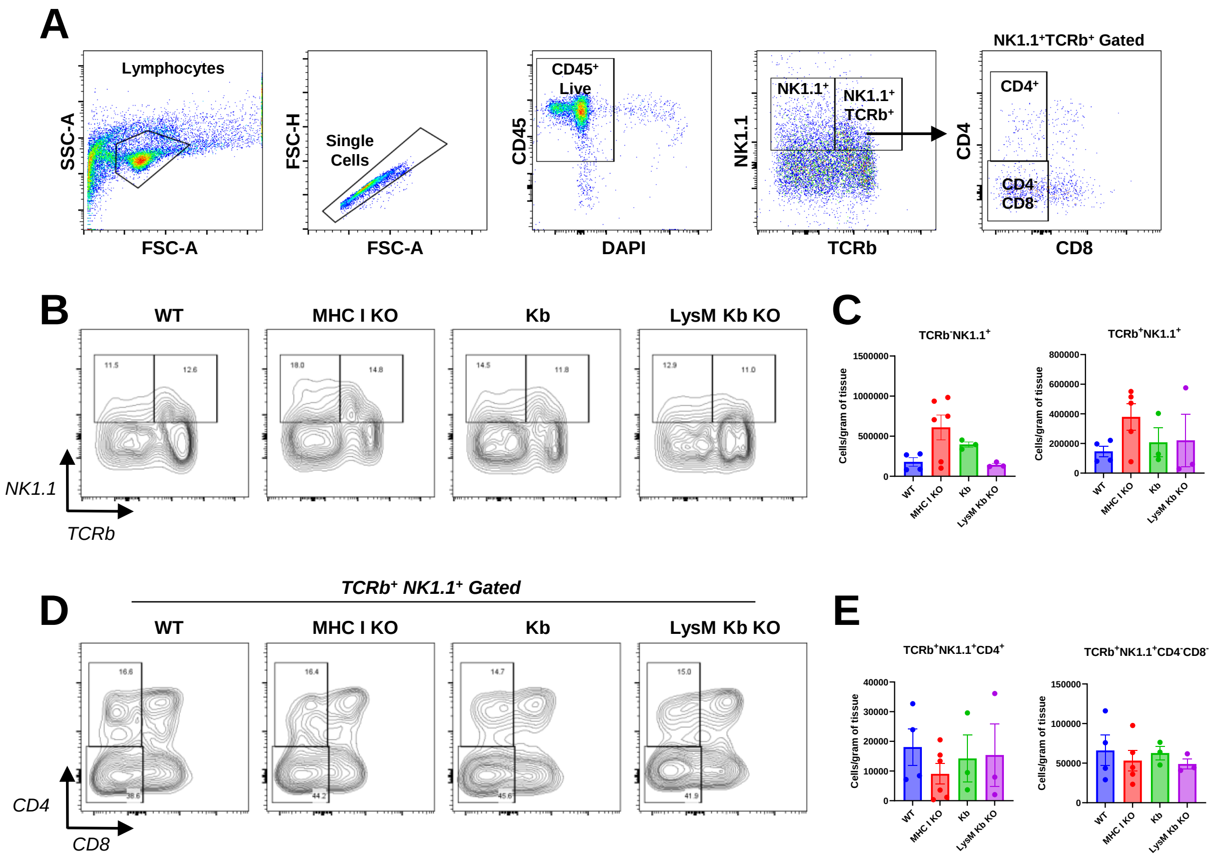
A
Lymphocytes
SSC-A
FSC-A
Single Cells
FSC-H
CD45+ Live
CD45
DAPI
NK1.1+
NK1.1+ TCRb+
NK1.1
TCRb
NK1.1+TCRb+ Gated
CD4+
CD4 CD8
CD4
CD8
B
WT
MHC I KO
Kb
LysM Kb KO
NK1.1
TCRb
C
TCRb-NK1.1+
TCRb+NK1.1+
Cells/gram of tissue
D
TCRb+ NK1.1+ Gated
WT
MHC I KO
Kb
LysM Kb KO
CD4
CD8
E
TCRb+NK1.1+CD4+
TCRb+NK1.1+CD4-CD8-
Cells/gram of tissue

Figure. S5. NK1.1+ and NK T cells are not impacted in livers of MHC I KO, Kb, or LysM Kb KO mice. Female WT, MHC I KO, Kb, and LysM Kb KO chow mice (n=3-6 per group). **(A)** Representative flow gating strategy for NK T cells. **(B-C)** Flow analysis of *NK1.1⁺ TCRb⁺* and *NK1.1⁺ TCRb⁻* cells. **(D-E)** Flow analysis of *CD4⁺ or CD4⁻CD8⁻* expression in *NK1.1⁺ TCRb⁺* cells. Data shown as the mean ± SEM. One-Way ANOVA was performed.

Figure S6

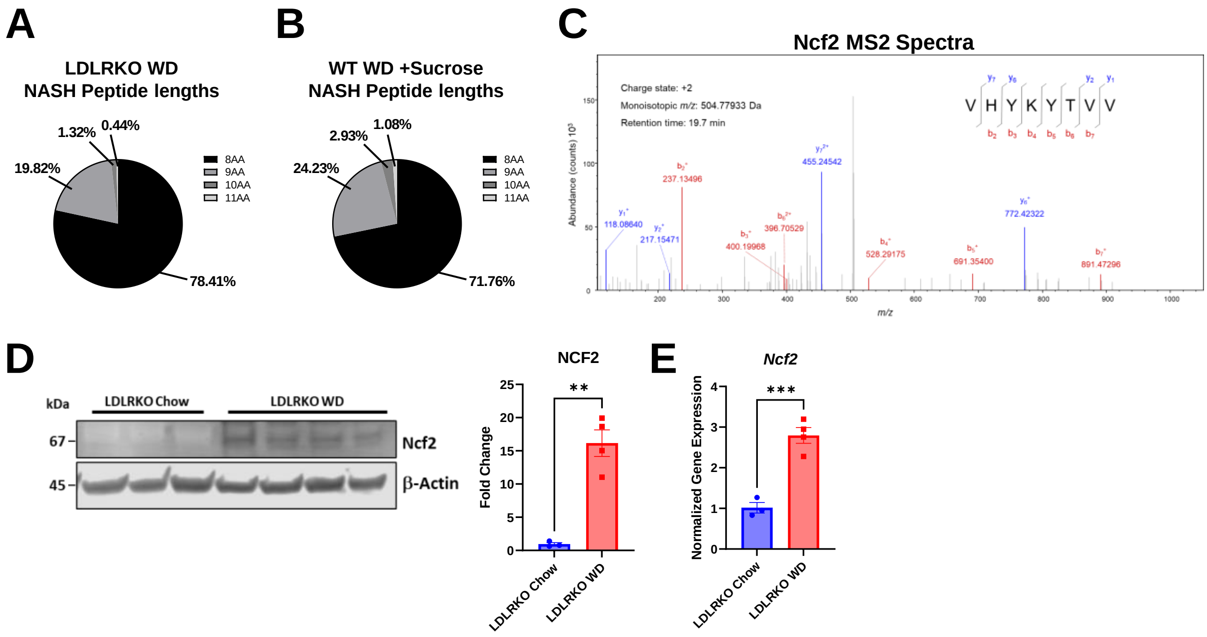

A
LDLRKO WD
NASH Peptide lengths
1.32%
0.44%
19.82%
78.41%
8AA
9AA
10AA
11AA
B
WT WD +Sucrose
NASH Peptide lengths
2.93%
1.08%
24.23%
71.76%
8AA
9AA
10AA
11AA
C
Ncf2 MS2 Spectra
Charge state: +2
Monoisotopic m/z: 504.77933 Da
Retention time: 19.7 min
V H Y K Y T V V
Abundance (counts) 10³
m/z
118.08640
217.15471
237.13496
400.19968
396.70529
455.24542
528.29175
691.35400
772.42322
891.47296
D
kDa
LDLRKO Chow
LDLRKO WD
67
45
Ncf2
β-Actin
NCF2
Fold Change
**
LDLRKO Chow
LDLRKO WD
E
Ncf2
Normalized Gene Expression
***
LDLRKO Chow
LDLRKO WD

Figure. S6. NASH Peptide characterization. LDLRKO mice on WD for 12 wks (n = 3-4 mice per group, 2 replicate cohorts). WT mice on chow or WD and sucrose water for 25 wks (n=5 mice per group, one cohort). **(A)** Unique NASH peptide lengths from LDLRKO NASH model **(A)** and WT NASH model **(B)**. **(C)** LC-MS/MS MS2 Ncf2 spectrum. Total liver Ncf2 protein **(D)** and gene **(E)** expression from LDLRKO NASH model. Data shown as the mean ± SEM. Two-tailed unpaired Student's t-tests was performed and considered statistically significant for $P<0.01$ (**) and $P<0.001$(***).

Figure S7

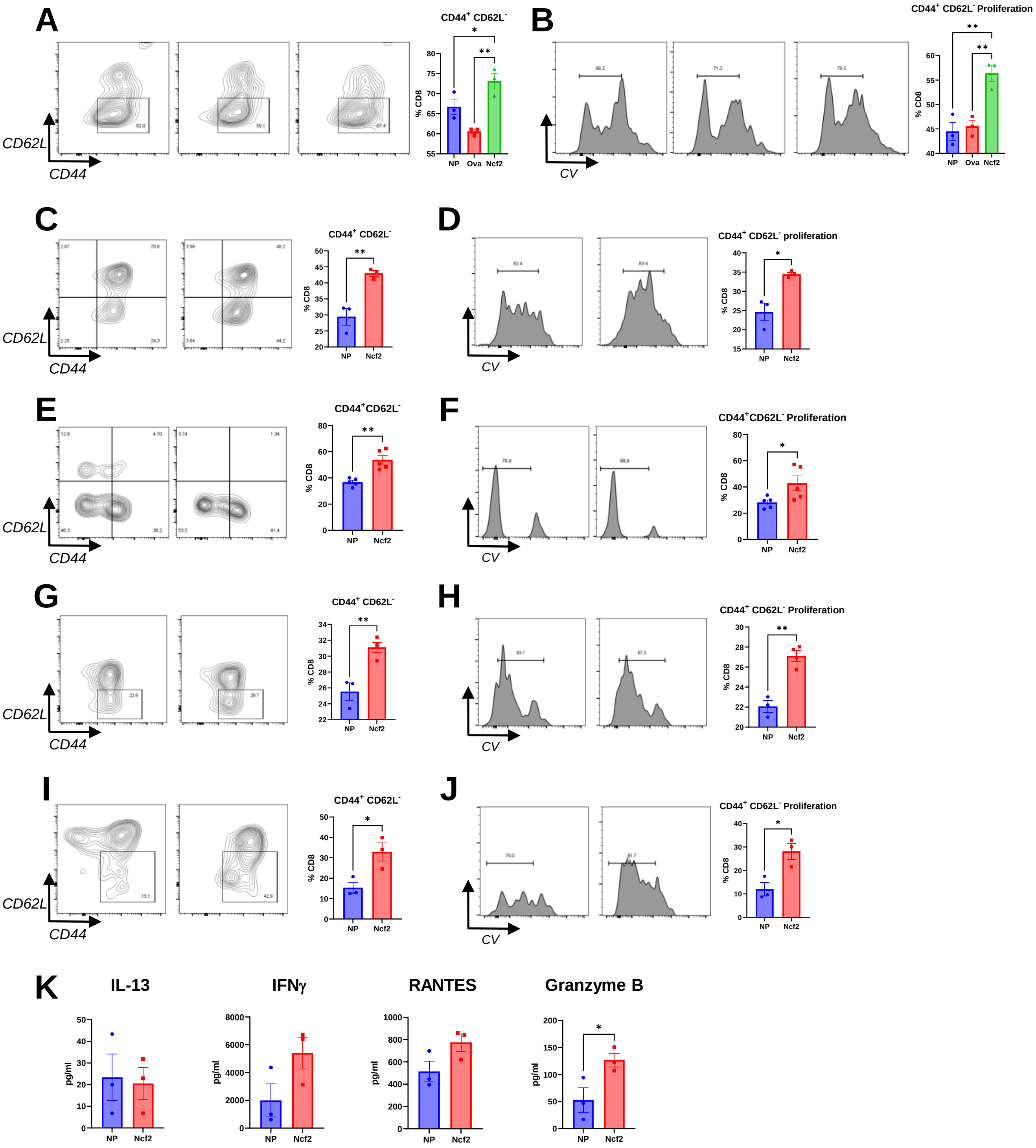
A
B
C
D
E
F
G
H
I
J
K
CD62L
CD44
CV
CD44+ CD62L-
CD44+ CD62L- Proliferation
% CD8
NP
Ova
Ncf2
IL-13
IFNγ
RANTES
Granzyme B
pg/ml

Figure. S7. Ncf2 peptide activates hepatic CD8$^+$ T cells and splenic CD8$^+$ T cells in various models of NASH. CD8$^+$ T cells were isolated from livers or spleens of Tac NASH, LDLRKO NASH, or WT NASH models (n = 2 mice combined per trial) done in 3-4 replicate studies and cocultured with NP, Ova, or Ncf2 pulsed RMA-S cells. Flow analysis of hepatic CD8$^+$ T cell activation **(A)** and proliferation **(B)** in the Tac NASH model after 3 days incubation with peptide pulsed RMA-S cells under stimulated (CD3/CD28) conditions. Flow analysis of splenic CD8$^+$ T cell activation **(C)** and proliferation **(D)** in the Tac NASH model after 5 days incubation with peptide pulsed RMA-S cells under stimulated conditions. Flow analysis of splenic CD8$^+$ T cell activation **(E)** and proliferation **(F)** in the Tac NASH model after 5 days incubation with peptide pulsed RMA-S cells under unstimulated conditions supplemented with 100ng/ml IL-2. Flow analysis of splenic CD8$^+$ T cell activation **(G)** and proliferation **(H)** in the WT NASH model after 5 days incubation with peptide pulsed RMA-S cells. Flow analysis of splenic CD8$^+$ T cell activation **(I)** and proliferation **(J)** in the LDLRKO NASH model after 5 days incubation with peptide pulsed RMA-S cells. **(K)** Cytokine analysis of media from splenic CD8$^+$ T cells incubated with Ncf2 peptide pulsed RMA-S cells in the LDLKO NASH model. Data shown as the mean ± SEM. Two-tailed unpaired Student's t-tests was performed for data sets with 2 groups and Two-way ANOVA was performed for groups more than 2 and was considered statistically significant for P<0.05 (*) and P<0.01 (**).
