# Supplementary material for: Myeloid cell MHC I expression drives CD8+ T cell activation in nonalcoholic steatohepatitis
Source: Front Immunol. 2024 Jan 11;14:1302006. doi: 10.3389/fimmu.2023.1302006 (PMC10808415; doi:10.3389/fimmu.2023.1302006)
Supplement: Supplementary file 2 [file Table_1.pdf]

TABLE S1. H&E PATHOLOGY SCORING

SAMPLE ID	Hepatocyte Necrosis	Oval Cell Hyperplasia	Lipid Accumulation	Lipid Vacuole Size	Glycogen	Comments
WT CHOW- 1	0	0	0	N/A	1	NSL
WT CHOW- 2	0	0	0	N/A	1	NSL
WT CHOW- 3	0	0	0	N/A	1	NSL
WT CHOW- 4	0	0	0	N/A	1	NSL
WT AMYLIN- 1	0	0	0	N/A	1	NSL
WT AMYLIN- 2	0	0	5	Mixed	N/A	Diffuse lipid accumulation, some minimal lymphocytic inflammation
WT AMYLIN- 3	0	0	5	Mixed	N/A	Diffuse lipid accumulation, some minimal lymphocytic inflammation
WT AMYLIN- 4	0	0	5	Mixed	N/A	Diffuse lipid accumulation, some minimal lymphocytic inflammation
WT AMYLIN- 5	0	0	5	Mixed	N/A	Diffuse lipid accumulation, some minimal lymphocytic inflammation
WT AMYLIN- 6	2	4	4	Macro	N/A	Multifocal yellow pigment in hepatocytes (presumed bile)
MHC I KO CHOW-1	0	0	0	N/A	0	NSL
MHC I KO CHOW-2	0	0	0	N/A	0	NSL
MHC I KO CHOW-3	0	0	0	N/A	0	NSL
MHC I KO CHOW-4	0	0	0	N/A	0	NSL
MHC I KO AMYLIN-1	0	0	4	Mixed	N/A	Diffuse lipid accumulation, some minimal lymphocytic inflammation
MHC I KO AMYLIN-2	0	0	4	Mixed	N/A	Diffuse lipid accumulation, some minimal lymphocytic inflammation
MHC I KO AMYLIN-3	0	0	4	Mixed	N/A	Diffuse lipid accumulation, some minimal lymphocytic inflammation
MHC I KO AMYLIN-4	1	0	5	Mixed	N/A	Diffuse lipid accumulation, some minimal neutrophilic and mononuclear inflammation
KB CHOW-1	0	0	0	N/A	1	Diffuse moderate glycogen accumulation
KB CHOW-2	1	0	0	N/A	1	Diffuse mild glycogen accumulation, neutrophilic inflammation associated with the focus of hepatocyte necrosis
KB CHOW-3	1	0	0	N/A	1	Diffuse mild glycogen accumulation, neutrophilic inflammation associated with the focus of hepatocyte necrosis
KB AMYLIN-1	2	1	2	Macro	0	Rare clusters of lymphocytes,and macrophages few neutrophils; multifocal yellow pigment in hepatocytes (presumed bile)
KB AMYLIN-2	0	0	5	Mixed	N/A	Diffuse lipid accumulation, some minimal lymphocytic inflammation
KB AMYLIN-3	2	4	0	N/A	N/A	Multifocal yellow pigment in hepatocytes (presumed bile)
LYSM KB KO CHOW-1	0	0	0	N/A	0	NSL
LYSM KB KO CHOW-2	1	0	0	N/A	0	Rare foci of neutrophilic/lymphocytic and histiocytic inflammation
LYSM KB KO CHOW-3	3	0	0	N/A	0	Lymphocytes, plasma cells and histiocytes with surrounding hepatocyte degeneration
LYSM KB KO CHOW-4	1	0	0	N/A	0	Rare clusters of lymphocytes, and histocytes with surrounding hepatocyte degeneration (hepatitis)

**LYSM KB KO AMYLIN-1**	0	0	5	Mixed	N/A	Diffuse lipid accumulation
**LYSM KB KO AMYLIN-2**	0	0	4	Micro	1	
**LYSM KB KO AMYLIN-3**	0	0	4	Mixed	1	
**LYSM KB KO AMYLIN-4**	0	0	4	Mixed	0	
