# Supplementary material for: Myeloid cell MHC I expression drives CD8+ T cell activation in nonalcoholic steatohepatitis
Source: Front Immunol. 2024 Jan 11;14:1302006. doi: 10.3389/fimmu.2023.1302006 (PMC10808415; doi:10.3389/fimmu.2023.1302006)
Supplement: Supplementary file 3 [file Table_2.pdf]

Table S2

Sequence	Length	Charge	MH+ [Da]	ΔM [ppm]	RT [min]	Proteins	Protein Accessions	Mascot IonScore	Sequest q-value	H2Kb binding Affinity(nM)
AALIYGKL	8	2	848.52402	-0.7	25.4304	1	Q9DC23		0.0070	47.49
AAYSYFRL	8	2	990.50434	0.1	29.6579	1	Q8R4U0		0.0070	2.83
AGPSRTVGL	9	2	857.48394	-0.29	18.168	1	Q64521		0.0037	860.84
AQYRFIYM	8	2	1107.52918	0.92	25.2868	2	P35235, P35235-2		0.0078	9.12
ATYSYKEAL	9	2	1045.52005	0.01	21.026	1	Q8R349		0.0037	29.22
AVHEFQNL	8	2	957.47886	-0.82	21.0445	1	Q8VCB3		0.0420	16.64
EAYRFTGL	8	2	956.48361	1.43	26.3243	2	Q8K015, Q8K015-2		0.0037	16.66
EIVSFQHL	8	2	972.51491	-0.59	27.935	1	Q8R502		0.0037	452.69
EPPVYANL	8	2	902.46181	0.71	29.7156	1	P05627		0.0037	4414.99
HVYYFAHL	8	2	1049.52033	1.38	25.9122	1	Q80SU7		0.0037	3.27
IFYYVQKL	8	2	1073.60299	-0.61	29.4592	1	Q69ZR2		0.0037	42.01
INLNYKDL	8	2	992.54112	-1.29	26.4768	1	Q9CY50		0.0490	49.34
INQIYEARV	9	2	1105.60003	-0.45	21.8794	1	Q8K2H4		0.0037	136.61
IPPEYRHL	8	2	1024.55744	-0.38	18.0785	7	P51125-4, P51125-7, P51125-6, P51125-5, P51125-1, P51125-3, P51125-2		0.0037	845.23
ISLDYQHL	8	2	988.50982	-0.81	27.4757	1	Q5SYL3		0.0057	7.82
ITFIFKSL	8	2	968.58153	0.76	34.9107	1	Q9JLN9		0.0044	3.3
KNIRFPLM	8	2	1018.58663	0.02	25.7934	6	Q80TF4-2, Q80TF4-5, Q80TF4-4, Q80TF4, Q80TF4-3, Q6ZPT1		0.0037	14.07
KNVTFHGI	8	2	915.50468	-0.12	18.1704	1	P19096		0.0037	89.31
KNYDFAQV	8	2	984.47852	-0.45	22.3666	3	Q8BGF7-2, Q8BGF7, Q8BGF7-3		0.0057	7.09
KSLEIRTL	8	2	959.58841	-0.68	19.3302	4	Q61301-2, Q61301-3, P26231, Q61301		0.0037	417.45
KTLEHINAI	9	2	1038.59422	-0.29	18.3673	1	P16301		0.0400	809.08
KVLEFERV	8	2	1019.58841	-0.64	21.0608	1	Q8CEC6		0.0037	136.24
QVVQFNRL	8	2	1003.56834	-1.39	24.1097	1	O88653		0.0037	62.22
RAFLFNKV	8	2	994.58326	-1.19	23.9096	1	P62700		0.0037	16.16
RSYNMPSL	8	2	967.46658	-0.8	23.6594	2	Q91ZV0, Q8R311		0.0037	31.18
SAARFFQL	8	2	939.50468	0.72	29.9437	1	Q9QY30		0.0037	10.13
SALIYSNL	8	2	880.47746	-0.06	32.1398	1	O55013		0.0037	4.45
SALKYYQL	8	2	985.53531	-0.79	25.0987	2	Q6ZPU9-1, Q6ZPU9-3		0.0037	10.19
SEYRYTLL	8	2	1044.53604	-0.45	27.2462	1	Q9D1Q6		0.0037	49.3
SGPDLSTAL	9	2	860.43599	-0.25	28.9577	1	Q91VS7		0.0340	1268.48
SNPEFRQL	8	2	990.50032	-0.34	20.6513	1	Q3UWM4		0.0078	50.52
SSAEFHSL	8	2	877.40502	-0.08	19.4898	1	Q61810-1		0.0037	16.85
SSVLYSRV	8	2	910.49926	-0.2	20.196	1	Q8CGC7		0.0037	14.48
SSYRFVQNV	9	2	1099.55308	0.39	22.4486	1	P42128		0.0044	4.2
STFVYNTM	8	2	978.42371	0.42	24.1463	1	Q8CFB4		0.0037	6.13
SVYTHSYL	8	2	969.46762	0.83	21.5721	5	Q3TZX8-3, Q3TZX8-2, Q8K0L2-2, Q8K0L2, Q3TZX8-1		0.0037	5.74
TIILFTKV	8	2	934.59718	-1.27	31.8743	2	Q8K2V6-2, Q8K2V6-1		0.0044	43.16
TNISFTNM	8	2	943.41896	0.3	24.212	1	Q9CYY7		0.0240	10.67
TNVLFNHL	8	2	957.51524	-0.57	27.9848	1	Q9D706		0.0037	16.43
TTYRFPEL	8	2	1026.52547	0.2	29.1235	2	Q8K440, Q8K440-2		0.0096	3.08
TVPELTQQM	9	2	1062.51359	0.12	22.6054	6	Q7TMM9, Q9D6F9, Q9ERD7, P68372, Q9CWF2, Q922F4		0.0044	3132.55
VFYEREVQM	9	2	1200.57177	0.35	24.5077	1	Q80SY3		0.0037	95.16
VGYRQPLV	8	2	931.53598	-0.76	21.0133	1	P97370		0.0046	137.01
VHYKYTVV	8	2	1008.55129	-0.22	18.6048	1	O70145		0.0037	40.94
VIFNYKGKNV	10	3	1181.66772	-0.38	19.3954	1	P14211		0.0037	424.81
VITNFSARI	9	2	1020.58366	0.61	25.3305	2	Q3UVL4-1, Q3UVL4-2		0.0037	48.12
VIVRFLTV	8	2	946.60841	-1.32	32.6811	1	P62245		0.0044	19.64
VMYRVIQV	8	2	1007.57065	0.02	26.3151	1	Q61069		0.0037	32.58
VNLVFEKI	8	2	961.57169	-0.82	30.0931	4	Q6AW69-4, Q6AW69-5, Q6AW69-1, Q6AW69-3		0.0037	51.25
VNRVFDKL	8	2	990.57309	-0.72	20.0245	1	P28076		0.0037	50.28
VNSIFQHL	8	2	957.51524	0.07	28.0498	1	Q80SU7		0.0037	16.64
VNVCYKEL	8	2	967.49173	0.27	28.3798	1	Q8BKT7		0.0037	33.28
VNYPRKIGA	9	2	1017.58399	-0.86	17.0715	1	O35488		0.0037	475.37
VQYEMRTL	8	2	1055.51901	-1.08	18.5278	1	Q80ZK0		0.0037	27.17
VSTKFEHL	8	2	960.51491	-0.03	17.972	1	B2RXC1		0.0037	27.81
VSYWFDQRF	9	2	1247.58438	0.3	34.0188	1	P54751		0.0037	15.32
VTITFKNM	8	2	953.51246	0.1	25.2831	2	Q8R4Y4, Q8R4Y4-2		0.0037	8.71
VVAEFGRI	8	2	890.50943	0.36	24.6606	1	Q8BXC6		0.0120	79.83
VVYIYRQI	8	2	1053.60914	1.19	25.6224	7	Q62417-3, Q62417-5, Q62417-2, Q62417-4, Q62417, Q62417-7, Q62417-6		0.0300	6.35
